# Identification of a novel strong promoter from the anhydrobiotic midge, *Polypedilum vanderplanki*, with conserved function in various insect cell lines

**DOI:** 10.1038/s41598-019-43441-x

**Published:** 2019-05-07

**Authors:** Yugo Miyata, Shoko Tokumoto, Yoichiro Sogame, Ruslan Deviatiiarov, Jun Okada, Richard Cornette, Oleg Gusev, Elena Shagimardanova, Minoru Sakurai, Takahiro Kikawada

**Affiliations:** 1 Center for Biological Resources and Informatics, Tokyo Institute of Technology, Yokohama, Japan; 2 Anhydrobiosis Research Group, Molecular Biomimetics Research Unit, Institute of Agrobiological Sciences, National Institute of Agriculture and Food Research Organization (NARO), Tsukuba, Japan; 3 Department of Integrated Biosciences, Graduate School of Frontier Sciences, The University of Tokyo, Kashiwa, Japan; 4 Department of Applied Chemistry and Biochemistry, National Institute of Technology, Fukushima College, Iwaki, Japan; 5 Institute of Fundamental Medicine and Biology, Kazan Federal University, Kazan, Russian Federation; 6 RIKEN-KFU Translational Genomics Unit, RIKEN Cluster for Science, Technology and Innovation Hub, RIKEN, Yokohama, Japan; 7 Preventive Medicine and Diagnosis Innovation Program, RIKEN Cluster for Science, Technology and Innovation Hub, RIKEN, Yokohama, Japan

**Keywords:** Transcription, Expression systems

## Abstract

Larvae of the African midge *Polypedilum vanderplanki* (Diptera: Chironomidae) show a form of extreme desiccation tolerance known as anhydrobiosis. The cell line Pv11 was recently established from the species, and these cells can also survive under desiccated conditions, and proliferate normally after rehydration. Here we report the identification of a new promoter, 121, which has strong constitutive transcriptional activity in Pv11 cells and promotes effective expression of exogenous genes. Using a luciferase reporter assay, this strong transcriptional activity was shown to be conserved in cell lines from various insect species, including S2 (*Drosophila melanogaster*, Diptera), SaPe-4 (*Sarcophaga peregrina*, Diptera), Sf9 (*Spodoptera frugiperda*, Lepidoptera) and Tc81 (*Tribolium castaneum*, Coleoptera) cells. In conjunction with an appropriate selection maker gene, the 121 promoter was able to confer zeocin resistance on SaPe-4 cells and allowed the establishment of stable SaPe-4 cell lines expressing the fluorescent protein AcGFP1; this is the first report of heterologous gene expression in this cell line. These results show the 121 promoter to be a versatile tool for exogenous gene expression in a wide range of insect cell lines, particularly useful to those from non-model insect species.

## Introduction

In fundamental and applied biological research, exogenous gene expression is one of the most commonly used and effective techniques. In basic research, the technique is used to clarify the functions of genes of interest through gain-of-function and loss-of-function experiments. In applied research, the most familiar example is industrial production of biological pharmaceuticals in microbial, mammalian, and insect cells. Exogenous gene expression is commonly driven by appropriate promoters, and their transcriptional activity is one of the critical factors governing the level of gene expression. Although several types of useful promoter have been developed for model organisms, the lack of suitable promoters is still a bottleneck in the study of non-model organisms.

Insect cell-based expression systems are used for commercial production of various veterinary and human vaccines^1,2^, and screening systems with insect cells are exploited for developing new insecticides^3^. The availability of such expression systems relies on the development of genetic engineering tools, but the development is often limited to specific cell lines. Although more than 900 insect cell lines have been established according to the ExPASy Cellosaurus database (http://web.expasy.org/cellosaurus), the research potential of most of these cell lines has not been fully explored due to a lack of suitable engineering tools. Versatile systems for heterologous gene expression in a variety of insect cells could enable deeper research in many of these cell lines, resulting in, for example, the development of cell lines with more efficient expression capability that yield larger amounts of target proteins.

Pv11 is a cultured cell line derived from embryos of the sleeping chironomid *Polypedilum vanderplanki*, which inhabits semi-arid regions in Africa, and which is the largest anhydrobiotic animal known^4,5^. Pv11 cells also display extreme desiccation tolerance; consequently, the cells can be stored in a dry state at room temperature, while retaining their ability to proliferate once rehydrated^6^. To improve our understanding of the molecular mechanisms underlying the anhydrobiotic ability, we have developed gene manipulation methods for Pv11 cells, and have identified the promoter of the *PvGapdh* gene as a constitutive promoter for heterologous gene expression in Pv11 cells^7^. However, the activity of this promoter depends on the nature of the downstream sequence: for example, when the mKeima-Red gene was placed under the control of the *PvGapdh* promoter, transfected Pv11 cells did not exhibit fluorescence as expected (Fig. S1). Therefore, to establish a workable heterologous overexpression system, the identification of a new constitutively strong promoter was necessary.

Pv11 cells have been used for a new dry preservation technique for proteins, in particular enzymes^8^. The activity of a desiccation-sensitive enzyme, luciferase (Luc), was preserved in dry Pv11 cells for up to 372 days. This could be due to the massive accumulation of anhydroprotectants in these cells: when *P*. *vanderplanki* larvae are subjected to dehydration, they accumulate several specific molecules such as trehalose, antioxidants and late embryogenesis abundant (LEA) proteins, which are believed to actively contribute to successful anhydrobiosis^5,9^. The corresponding anhydroprotectant coding genes are also upregulated in Pv11 cells undergoing the pretreatment that induces anhydrobiosis^10^. However, the molecular biological evidence for the function of these molecules in anhydrobiosis remains to be elucidated. A powerful heterologous expression system would allow the function of relevant genes to be extensively tested in anhydrobiotic Pv11 cells.

Here we describe the identification and characterization of a novel strong promoter, named 121, from the *P*. *vanderplanki* genome. A luciferase reporter assay showed the extremely high transcriptional activity of this promoter in Pv11 cells. Furthermore, the 121 promoter was also highly active in S2 (*Drosophila melanogaster*, Diptera), SaPe-4 (*Sarcophaga peregrina*, Diptera), Sf9 (*Spodoptera frugiperda*, Lepidoptera) and Tc81 (*Tribolium castaneum*, Coleoptera) cells. In addition, we showed the evidence of the successful establishment for stable AcGFP1-expressing SaPe-4 cells using the 121 promoter, and this is the first report of exogenous gene expression and its application in SaPe-4 cells. Thus, these results indicate the versatility of the 121 promoter in a wide range of insect cell lines, and provide a new and powerful option for the expression of exogenous genes in non-model insect cell lines.

## Results

### A promoter with constitutively strong transcriptional activity in Pv11 cells

**Figure 1:**
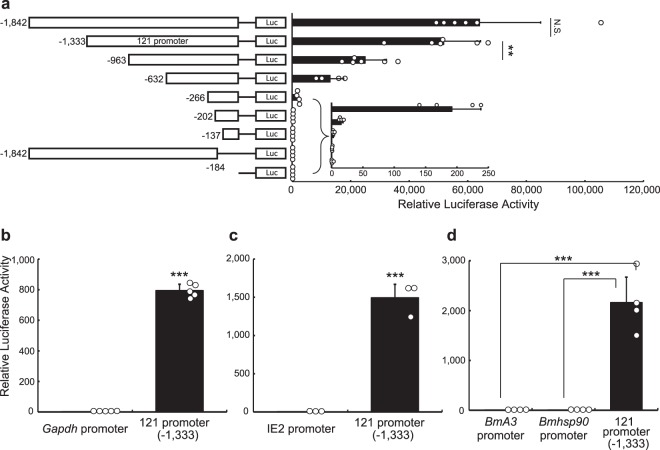
Transcriptional activity of the 121 promoter in Pv11 cells. A scheme of luciferase expression constructs with different fragments of the *Pv*.*00443* gene promoter region is shown on the left and relative values are shown on the right. The inset shows a magnification of relative luciferase activity for low activity constructs (**a**). Comparison of the relative luciferase activity in Pv11 cells between the 121 promoter (−1,333) and the *PvGapdh* promoter (**b**), the OpIE2 promoter (**c**) and the *BmA3* and *Bmhsp90* promoters (**d**). Normalized values are expressed as mean ± standard deviation (SD). **p < 0.01; ***p < 0.001; N.S., not significant; n = 3–6 in each group.

To isolate a strong constitutive promoter in *P*. *vanderplanki*, we initially searched for a gene that is constitutively and highly expressed in *P*. *vanderplanki* larvae. According to the transcriptome data from a *P*. *vanderplanki* genome database, MidgeBase (http://bertone.nises-f.affrc.go.jp/midgebase/)^11^, the *Pv*.*00443* gene located on scaffold 121 is constitutively and highly expressed during both dehydration and rehydration in *P*. *vanderplanki* larvae (Fig. S2); however, the function of the gene is completely unknown (http://bertone.nises-f.affrc.go.jp/midgebase/pv_gene_page/Pv.00443.html). We cloned a 1,842 bp genomic fragment from the putative transcriptional start site of *Pv*.*00443*, where we expected its promoter to reside (Fig. 1a; the DNA sequence is shown in Supplementary Data 1), and confirmed it to have strong transcriptional activity in Pv11 cells (Fig. S3). To identify the minimal length of this fragment necessary for strong transcriptional activity, we constructed eight deletion mutants and assessed their promoter activity with a luciferase assay system (Fig. 1a). As shown in Fig. 1a, a 1,333bp-fragment (−1,333) retained the maximum promoter activity of the original 1,842bp-fragment (−1,842), while shorter fragments exhibited reduced activity. Therefore, we named the 1,333bp-fragment the ‘121 promoter.’

We compared 121 promoter activity with that of the *PvGapdh* promoter, which we cloned previously^7^. Figure 1b shows that the activity of the 121 promoter is much higher than that of the *PvGapdh* promoter in Pv11 cells. We next compared 121 promoter activity with that of the commercially available OpIE2 insect promoter, which is derived from the *Orgyia pseudotsugata* multicapsid nuclear polyhedrosis virus^12,13^, as well as the well-known lepidopteran promoters, *BmA3* and *Bmhsp90*^*P2*.*9k*^, which are also active in dipteran and coleopteran cell lines^14^. We observed that the 121 promoter had a much higher transcriptional activity in Pv11 cells than the OpIE2, *BmA3* and *Bmhsp90* promoters (Fig. 1c,d).

### Conserved activity of the 121 promoter in a variety of insect cell lines

**Figure 2:**
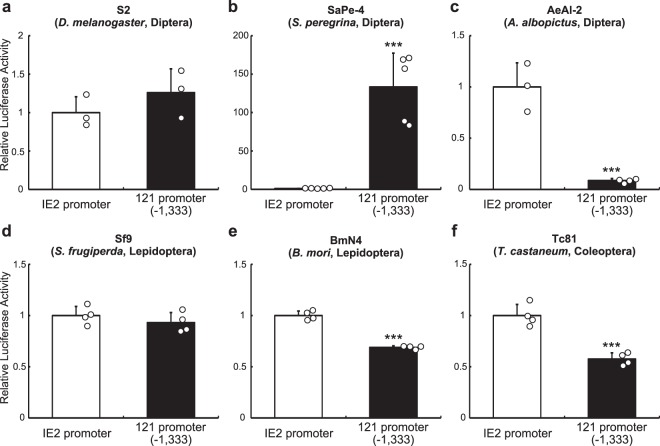
Transcriptional activity of the 121 promoter in various insect cell lines. The baculovirus promoter, OpIE2, and the 121 promoter (−1,333) were ligated to a luciferase reporter gene, and the plasmid vectors were transfected into various insect cells: S2 (**a**), SaPe-4 (**b**), AeAl-2 (**c**), Sf9 (**d**), BmN4 (**e**) and Tc81 (**f**). Forty-eight hours after transfection, luciferase activity was measured and normalized to secreted embryonic alkaline phosphatase (SEAP) activity. Normalized values are expressed as mean ± SD. *** for *p* < 0.001; n = 3–5 in each group.

The substantial transcriptional activity associated with the 121 promoter led us to examine its transcriptional activity in cell lines derived from other insect species, i.e. S2 (*D*. *melanogaster*), Sape-4 (*Sa*. *peregrina*), AeAl-2 (*Aedes albopictus*), Sf9 (*Sp*. *frugiperda*) BmN4 (*Bombyx mori*) and Tc81 (*T*. *castaneum*). As shown in Figs 2 and S4, the 121 promoter exhibited transcriptional activity in all cell lines tested. Intriguingly, the OpIE2 promoter was much less effective than the 121 promoter in Sape-4 cells and Pv11 cells (Figs 2b and S4). Conversely, in AeAl-2, the OpIE2 promoter showed higher activity than the 121 promoter, however the 121 promoter still exhibited transcriptional activity (Figs 2c and S4). In S2 and Sf9 cells, both promoters exhibited similar levels of activity, while in BmN4 and Tc81 cells, the 121 promoter had a slightly lower activity than the OpIE2 promoter. Together, these data suggest that the 121 promoter can exert high transcriptional activity in most insect cell lines.

**Figure 3:**
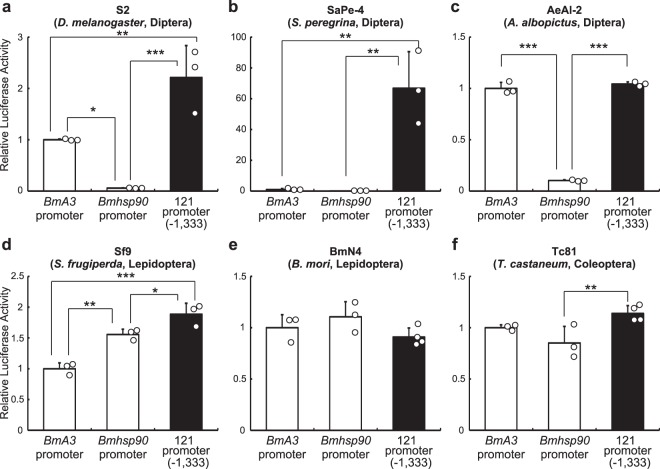
Comparison of the transcriptional activity of the 121 promoter with the strong lepidopteran promoters, *BmA3* and *Bmhsp90*. The *Bombyx mori* promoters and the 121 promoter (−1,333) were ligated to a luciferase reporter gene, and the plasmid vectors were transfected into various insect cells: S2 (**a**), SaPe-4 (**b**), AeAl-2 (**c**), Sf9 (**d**), BmN4 (**e**) and Tc81 (**f**). Forty-eight hours after transfection, luciferase activity was measured and normalized to SEAP activity. Normalized values are expressed as mean ± SD. *p < 0.05; **p < 0.01; ***p < 0.001; n = 3–4 in each group.

When compared to the *BmA3* and *Bmhsp90* promoters, the 121 promoter was much more active in Sape-4 cells (Fig. 3b), and also showed higher transcriptional activity in S2 and Sf9 cells (Fig. 3a,d). In AeAl-2 and Tc81 cells, the 121 promoter displayed similar transcriptional activity to that of the *BmA3* promoter, but was more active than the *Bmhsp90* promoter (Fig. 3c,f). In BmN4 cells, there was no significant difference among the three promoters (Fig. 3e). Taken together, the results show that the 121 promoter has stronger or similar promoter activity to that of silkworm promoters in insect cell lines.

### Application of the 121 promoter in SaPe-4, a non-model insect cell line

**Figure 4:**
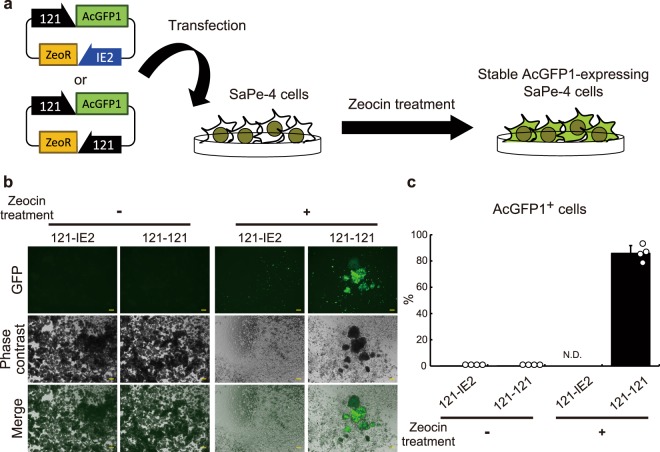
Use of the 121 promoter for stable expression in SaPe-4. The experimental scheme is shown in (**a**). To establish a stable cell line that expresses AcGFP1 under control of the 121 promoter, the 121 promoter (−1,333) was ligated to the ZeoR gene, and the OpIE2 promoter was used as a control. The plasmid vectors were transfected into SaPe-4 cells and transformed cells were selected by addition of zeocin to the growth medium. After three weeks, images of the cells were acquired using a BZ-X700 microscope. Merged images are shown at the bottom. Zeocin-untreated samples are shown on the left as a control (**b**). The proportions of AcGFP1^+^ cells in the live cell population (%) were analyzed using a CytoFLEX S flow cytometer after three weeks in culture with or without zeocin (**c**). Scale bars, 200 µm. The values are expressed as mean ± SD. N.D. means that sufficient cells for analysis were not detected (ref. Figs S5 and S6); n = 4 in each group.

To the best of our knowledge, no system for heterologous gene expression has yet been developed in SaPe-4 cells, whose original species (fleshfly) produces antimicrobial factors with therapeutic potency against cancer and cataract^15–17^. Figures 2b and S4 show that the 121 promoter can drive ectopic expression of luciferase and TagRFP in SaPe-4 cells; this is the first description of a successful overexpression system in SaPe-4. To further examine possible use of the 121 promoter in SaPe-4, we attempted to establish a stable cell line with zeocin resistance. Expression cassettes for AcGFP1 and zeocin-resistance (ZeoR) genes were constructed using a commercially available plasmid, pIZ (Thermo Fisher Scientific), as a platform. The resulting vectors were named ‘121-IE2’ and ‘121-121’; both of them drive AcGFP1 gene expression under control of the 121 promoter, while the ZeoR gene is governed by either the OpIE2 or the 121 promoter, respectively (Fig. 4a; the same naming convention is applied to similar constructs hereinafter). The plasmids were transfected into Sape-4 cells, which were then treated with 100 µg/ml zeocin. After three weeks, most surviving cells expressed AcGFP1 in the 121-121-transfected group, while most cells were dead in the 121-IE2-transfected group (Figs 4b,c, S5 and S6). These results suggest that the 121 promoter has significant potential to provide a new, alternative and efficient method of expressing exogenous genes, and for the establishment of stable transformed cells, in non-model insect cell lines such as Sape-4.

### Use of the 121 promoter for stable expression in other insect cells

**Figure 5:**
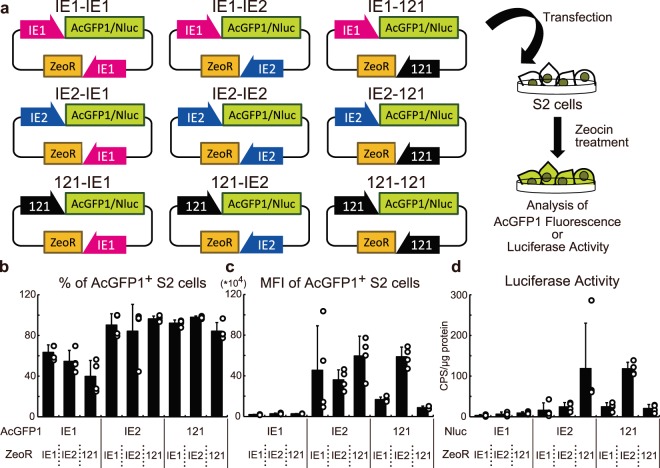
Use of the 121 promoter for the establishment of stably transfected S2 cells. The experimental scheme is shown in (**a**). Nine plasmid vectors for the expression of AcGFP1/Nluc and ZeoR were transfected into S2 cells. After zeocin selection, the cells were assessed for AcGFP1 fluorescence or luciferase activity. In the AcGFP1-expressing cells, the proportions and MFIs of AcGFP1^+^ cells in the live cell population were analyzed by a CytoFLEX S flow cytometer (**b**,**c**). In the Nluc-expressing cells, the luciferase activities were measured (**d**). The values are expressed as mean ± SD; n = 3–4 in each group. Statistical analysis is presented in Tables 1–4.

The baculovirus promoters, OpIE2 and OpIE1, have been commonly used for the establishment of stable cell lines expressing heterologous genes in several insect cell lines^18^. Because our data show that the 121 promoter exhibits strong activity in various insect cell lines (Figs 2, 3 and S4), we hypothesized that it might allow easier establishment of stably transfected cells than the baculovirus promoters. To test this, we constructed nine plasmid vectors for AcGFP1 or NanoLuc® Luciferase (Nluc) expression (Fig. 5a).

S2 cells transfected with the plasmid vectors were treated with zeocin for three weeks, and then AcGFP1 fluorescence or luciferase activity was measured (Figs 5 and S7 and Tables 1 and S1). In the AcGFP1 experiment, use of the 121 promoter did not show any improvement in the percentage of AcGFP1^+^ cells and mean fluorescent intensities (MFIs) compared to the IE2-IE1-, and IE2-IE2-transfected groups (Fig. 5b,c; Tables 2 and 3). However, in the luciferase experiment, IE2-121-transfected cells showed significantly higher luciferase activity than the IE2-IE1- and IE2-IE2-transfected groups (Fig. 5d; Table 4). Furthermore, a significant difference was observed between the IE2-IE1- and 121-IE2-transfected groups, and there was a marginal difference between IE2-IE2 and 121-IE2 groups (p = 0.0503; Table 4). Taken together, transfecting IE2-121 and 121-IE2 plasmid vectors generated the cells with the maximum AcGFP1 and Nluc expression, and these results suggest that the combination of the 121 and the OpIE2 promoters functions best as a stable expression vector system in S2 cells.

**Table 1:** Two-way ANOVA in Fig. 5b–d.

Cell	Data Source (Dependent Variable)	Source of Variation	% of total variation	*p-*value
S2	Figure 5b (% of AcGFP1^+^ cells)	Interaction	7.777	0.0841
		AcGFP1	66.9	<0.0001
		ZeoR	2.543	0.2396
	Figure 5c (MFI of AcGFP1^+^ cells)	Interaction	23	0.0023
		AcGFP1	45.54	<0.0001
		ZeoR	3.182	0.237
	Figure 5d (Luciferase activity)	Interaction	37.66	0.0009
		Nluc	15.59	0.0107
		ZeoR	8.897	0.0621

**Table 2:** Statistical analysis of percentages of AcGFP1^+^ S2 cells in Fig. 5b.

	IE1-IE1	IE1-IE2	IE1-121	IE2-IE1	IE2-IE2	IE2-121	121-IE1	121-IE2	121-121
IE1-IE1		ns	ns	ns	ns	*	ns	*	ns
IE1-IE2	—		ns	**	*	**	**	***	*
IE1-121	—	—		***	***	***	***	***	***
IE2- IE1	—	—	—		ns	ns	ns	ns	ns
IE2- IE2	—	—	—	—		ns	ns	ns	ns
IE2- 121	—	—	—	—	—		ns	ns	ns
121- IE1	—	—	—	—	—	—		ns	ns
121- IE2	—	—	—	—	—	—	—		ns
121- 121	—	—	—	—	—	—	—	—	

Statistical analysis was performed by Tukey test as a post-hoc test for two-way ANOVA.

*p < 0.05; **p < 0.01; ***p < 0.001; ns, not significant.

**Table 3:** Statistical analysis of MFIs of AcGFP1^+^ S2 cells in Fig. 5c.

	IE1-IE1	IE1-IE2	IE1-121	IE2-IE1	IE2-IE2	IE2-121	121-IE1	121-IE2	121-121
IE1-IE1		ns	ns	*	ns	**	ns	**	ns
IE1-IE2	—		ns	*	ns	**	ns	**	ns
IE1-121	—	—		*	ns	*	ns	**	ns
IE2- IE1	—	—	—		ns	ns	ns	ns	ns
IE2- IE2	—	—	—	—		ns	ns	ns	ns
IE2- 121	—	—	—	—	—		*	ns	**
121- IE1	—	—	—	—	—	—		*	ns
121- IE2	—	—	—	—	—	—	—		**
121- 121	—	—	—	—	—	—	—	—	

Statistical analysis was performed by Tukey test as a post-hoc test for two-way ANOVA.

*p < 0.05; **p < 0.01; ns, not significant.

**Table 4:** Statistical analysis of luciferase activities of AcGFP1^+^ S2 cells in Fig. 5d.

	IE1-IE1	IE1-IE2	IE1-121	IE2-IE1	IE2-IE2	IE2-121	121-IE1	121-IE2	121-121
IE1-IE1		ns	ns	ns	ns	**	ns	**	ns
IE1-IE2	—		ns	ns	ns	*	ns	*	ns
IE1-121	—	—		ns	ns	*	ns	*	ns
IE2-IE1	—	—	—		ns	*	ns	*	ns
IE2-IE2	—	—	—	—		*	ns	^†^	ns
IE2-121	—	—	—	—	—		*	ns	*
121-IE1	—	—	—	—	—	—		ns	ns
121-IE2	—	—	—	—	—	—	—		*
121-121	—	—	—	—	—	—	—	—	

Statistical analysis was performed by Tukey test as a post-hoc test for two-way ANOVA.

^†^*p* = 0.0503; *p < 0.05; **p < 0.01; ns, not significant.

Next, we tested the nine plasmid vectors in Sf9 cells and, as above, measured AcGFP1 fluorescence or luciferase activity in the transfected cells after three weeks of zeocin treatment (Fig. S7 and Table S1). In the AcGFP1 experiment, the IE2-121-transfected cells showed a higher percentage of AcGFP1^+^ cells than the IE2-IE1- and IE2-IE2-transfected groups (Table S2), and a higher MFI in AcGFP1^+^ cells than the IE2-IE2-transfected group (Table S3). In the luciferase experiment, IE2-121-transfected cells showed higher luciferase activity than IE2-IE2-transfected cells. However, the 121-IE2-transfected group performed much less than the IE2-IE2- or IE2-121-transfected cells (Table S4). Thus, IE2-121 is the best construct in Sf9 cells, rather than the reversed combination of the two promoters.

## Discussion

Although the establishment of the Pv11 cell line was reported in 2010^4^, no practical system for exogenous gene expression in Pv11 cells has been available until recently. The present study identified a strong promoter, the 121 promoter, from the *P*. *vanderplanki* genome, which showed very strong activity not only in Pv11 cells but also in S2, SaPe-4 and Sf9 cells. Furthermore, the 121 promoter expression system conferred resistance to zeocin and the ability to stably express AcGFP1 in SaPe-4 cells. Our results demonstrate the versatility of the 121 promoter in a wide range of insect cell lines, and provide an alternative to the OpIE2 promoter for exogenous gene expression in non-model insect cell lines.

Using a series of deletions (Fig. 1), we were able to define 121 promoter variants with different levels of transcriptional activity. The 963 bp and 632 bp fragments showed 50% and 25% of the activity of the full-length 121 promoter, respectively (5,175 ± 1,260 relative luciferase activity (RLA) for the 121 promoter; 2,491 ± 729 RLA for the 963 bp fragment; 1,293 ± 465 RLA for the 632 bp fragment). This is important, because it allows the use of different strength promoters such that the resulting protein expression levels can be controlled according to the requirements of a particular experiment. In addition to these medium-strength promoters, the shorter 266 bp, 202 bp and 137 bp fragments are candidates for a minimal promoter in Pv11 cells. The availability of minimal promoters will be useful in developing further experimental systems, for example, for inducible gene epression^19^ and for genome-wide enhancer screening^20,21^; this is one strategy for unveiling the gene network that underpins anhydrobiosis in *P*. *vanderplanki*.

The identification of the 121 promoter could facilitate the clarification of molecular mechanisms underlying anhydrobiosis in Pv11 cells, thanks to its high transcriptional activity. For example, other workers have used exogenous expression of a variety of fluorescent sensor proteins to reveal molecular events and signals under several conditions *in vivo* and *in vitro*^22–25^. In addition, forced expression of tagged proteins can be used to assess specific protein–protein and protein–DNA interactions^26–28^. Heterologous gene expression systems can also be utilized for both gain-of-function and loss-of-function experiments by exploiting, for example, the CRISPR/Cas9 system for genome modification^29,30^. In our forthcoming research, we plan to use the 121 promoter and its variants to make these cutting-edge molecular biological technologies available for Pv11 cells and thereby to unveil the mechanisms of anhydrobiosis.

In the fleshfly, *Sa*. *peregrina*, a large number of antimicrobial factors have been identified as components of the immune system, and these have potential for various medical applications^31^. For example, 5-S-glutathionyl-N-β-alanyl-3,4-dihydroxyphenylalanine is an antimicrobial substance^32^ with free radical scavenging activity^33^; as a result of this activity, it inhibits tumor growth^15,16^, progression of cataract^17^ and apoptosis in neuronal cells^34^. However, the precise functions or activation mechanisms of many other antimicrobial peptides remain to be elucidated^35–37^. Our present work clearly showed the effectiveness of the 121 promoter in SaPe-4 cells (Figs 2b, 3b, 4 and S4), and therefore this promoter will make molecular biological experiments possible in SaPe-4 cells. This experimental approach with cell cultures allows high-throughput screening and will surely contribute to the characterization of currently uncharacterized immune molecules from *Sa*. *peregrina* and other insects, some of which molecules will have medical or biotechnological utility.

We examined the suitability of the 121 promoter for use in a stable expression system in S2 and Sf9 cells (Figs 5 and S7). In S2 cells, the combination of the 121 and OpIE2 promoters yielded the highest values of both percentage and MFI of AcGFP1^+^ cells, as well as of luciferase activity (Fig. 5 and Tables 1–4). In Sf9 cells, although IE2-121-transfected cells exhibited the highest values of these parameters in AcGFP1- and luciferase-expressing cells, 121-IE2-transfected cells did not perform better than IE2-IE1- and IE2-IE2-transfected cells (Fig. S7 and Tables S1–4). The difference in effectiveness of the promoters in S2 and Sf9 cells may be attributed to the distinct origin of each cell line. Sf9 cells were derived from a lepidopteran species (*Sp*. *frugiperda*), while S2 cells were established from the fruit fly, *D*. *melanogaster*, which is classified within Diptera, the same order as *P*. *vanderplanki*. Given that SaPe-4 cells also originate from a dipteran species, and that the 121 promoter is highly active in SaPe-4 cells, it seems likely that the strength of 121 promoter activity will depend on the evolutionary relatedness of the recipient cell type to *P*. *vanderplanki*.

There is a discrepancy of the 121 promoter activity between the transient and stable expression experiments of Sf9 cells (Figs 2d and S7); in the transient expression experiment, the 121 promoter showed similar levels of the transcriptional activity to the OpIE2 promoter (Fig. 2d), while in the stable ones, the 121 promoter activity was lower than that of the OpIE2 promoter (Fig. S7). These results mean that the 121 promoter on the plasmid vectors is highly activated, while the promoter activity is decreased when the 121 promoter is inserted into the Sf9 genome. The cause of the reduced promoter activity may be lower compatibility of the 121 promoter sequence with *Sp. frugiperda* genome. Numerous studies showed the epigenetic modification, like methylation, on exogenously integrated promoters and the suppressive effect on the promoter activities^38–41^. Further, the difference of the related molecules or DNA methylation pattern was suggested between Lepidoptera and Diptera including *D*. *melanogaster*^42–44^. Taken together, the pattern of the DNA methylation might play a critical role on the transcriptional activity of the 121 promoter in the stable expression system.

To gain insight into the functional makeup of the 121 promoter, we searched its sequence for potential transcription factor binding sites (TFBSs; Fig. S8). The promoter sequence was scanned for known insect TFBS motifs (JASPR insects 2016) using the software tool, FIMO^45^, and the best matches were annotated. Comparison of the annotated sequence with the level of luciferase activity recorded for each promoter fragment in the deletion experiment (Fig. 1a) identified TFBSs of three transcription factors, nubbin (nub), forkhead (fkh) and hunchback (hb), whose presence appeared to correlate with 121 promoter activity. Phylogenetic analysis of the three transcription factors showed four clusters in total; we observed a notable point of difference between the two clusters, nubbin #1 and nubbin #2: while nubbin #1 contained proteins from all investigated insect species, nubbin #2 did not contain examples from *Aedes* species. This result may be relevant to the very low activity of the 121 promoter in AeAl-2 (Fig. 2c), and we hypothesized that nubbin variants and the corresponding binding site play a critical role in determining 121 promoter activity. However, when we compared the transcriptional activity of the 121 promoter with or without a nubbin binding site, there was no difference (Fig. S8 and Table S5). Therefore, the exact mechanism regulating 121 promoter activity remains to be investigated.

In conclusion, the 121 promoter displays strong transcriptional activity in Pv11 cells, as well as in distantly related insect cell lines. This promoter will allow advanced molecular engineering, not only in Pv11 cells, where it will be instrumental in further characterizing the mechanisms underlying anhydrobiosis, but also in a large array of insect cells for various applications.

## Materials and Methods

### Cell culture

Pv11 cells were originally established in our lab^4^. AeAl-2, Sape-4 and Tc81 cells were obtained as file numbers ANJP 1715, ANJP 1784 and ANJP 1805, respectively, from the GeneBank of NARO (https://www.gene.affrc.go.jp/index_j.php). BmN4 cells were gratefully received from Prof. Toru Shimada (Univ. Tokyo). S2 and Sf9 cells were purchased from Thermo Fisher Scientific, Waltham, MA, USA. Pv11, AeAl-2 and BmN4 cells were cultured in IPL-41 Insect Medium (Thermo Fisher Scientific) supplemented with 2.6 g/L Bacto™ Tryptose Phosphate Broth (Thermo Fisher Scientific), 10% (v/v) fetal bovine serum (FBS), and 0.05% (v/v) of an antibiotic and antimycotic mixture (penicillin, amphotericin B, and streptomycin; Merck, Darmstadt, Germany)^7^. S2 and Sf9 cells were cultured in Schneider’s *Drosophila* Medium (Thermo Fisher Scientific) plus 10% (v/v) FBS, and Sf900III medium (Thermo Fisher Scientific), respectively. Sape-4 and Tc81 cells were cultured in Grace’s Insect Medium (Thermo Fisher Scientific) with 10% (v/v) FBS.

### Expression vectors for transient expression

To isolate a strong constitutive promoter, the promoter region of the *Pv*.*00443* gene was cloned after reference to the RNA-seq data in MidgeBase (http://bertone.nises-f.affrc.go.jp/midgebase/)^11^. AcGFP1-expressing vectors containing a deletion series of the 121 promoter (Fig. S3) were constructed by replacing the *PvGapdh*-promoter region of pPGK-AcGFP1^7^ with the 121 promoter series (pP121K-AcGFP1 series; Fig. S3). The AcGFP1 genes of the 121 promoter vector series were replaced with the MetLuc gene from pMetLuc2 (TaKaRa Bio, Kusatsu, Shiga, Japan) to construct MetLuc-expression vectors for the deletion experiment in Fig. 1a. The promoter regions of the MetLuc-expression vectors were replaced with the OpIE2, *BmA3* and *Bmhsp90* promoters (Figs 1–3) from pIZ (Thermo Fisher Scientific), pGL3-A3 and pGL3-hsp90^P2.9k14^, using the SLiCE method^46^ or NEBuilder® HiFi DNA Assembly Master Mix (New England BioLabs, Ipswich, MA, USA). The AcGFP1 gene of pPGK-AcGFP1 was replaced with the MetLuc gene for the comparison of promoter activity between the 121 and *PvGapdh* promoters (Fig. 1b). The SEAP gene was cloned from pSEAP2 (TaKaRa Bio) and inserted in place of the MetLuc gene in the expression vectors with the *PvGapdh*, OpIE2 and *Bmhsp90* promoters.

### Expression vectors for stable expression

The construction scheme of the vectors for stable expression is presented in Figs S9 and S10. First, we newly created a basic vector with two gene cassettes: ‘121 promoter-MCS’ and ‘121 promoter-ZeoR’ (the complete sequence is shown in Supplementary Data 2). For construction of the vectors, the fragments were prepared by PCR (Table S6) and ligated by NEBuilder® HiFi DNA Assembly: the AmpR gene from pIREShyg3 (TaKaRa Bio); the 121 promoter from pP121K-AcGFP1; pUC ori, ZeoR and OpIE2 polyadenylation site from pIZ (Thermo Fisher Scientific). For AcGFP1 expression, the basic vector was digested with *Hin*dIII and *Xho*I, and the AcGFP1 fragment, prepared by PCR, was inserted using a NEBuilder® HiFi DNA Assembly kit. For the remaining constructs, the backbone vectors and insert fragments were prepared by enzyme digestion and PCR, respectively, and HiFi assembly was carried out.

To construct the Nluc expression vectors, the AcGFP1 gene in the nine vectors was replaced by the Nluc gene by NEBuilder® HiFi DNA Assembly; the backbone vectors were prepared by *Hin*dIII and *Xho*I digestion, and the PCR fragment of Nluc gene from pNL1.1 (Promega,  Fitchburg, WI, USA) was ligated into the cut vectors.

### Transfection and luciferase reporter assay for transient expression

Transfection into Pv11 cells was carried out using a NEPA21 Super Electroporator (Nepa Gene, Ichikawa, Chiba, Japan) as described previously^7^. S2, SaPe-4, AeAl-2, Sf9 and BmN4 cells were seeded at a density of 5.0 × 10^5^ cells per well in 6-well plates. The next day, 0.75 µg MetLuc-reporter vector and 0.75 µg SEAP-reference vector were transfected with 1.25 µl FuGENE6 (Promega). The medium was collected 72 h later, and luciferase activity was measured using an ARVO luminometer (PerkinElmer, Waltham, MA, USA) with the Ready-To-Glow dual secreted reporter assay system (TaKaRa Bio). For Tc81 transfection, 10 µg MetLuc-reporter vector and 10 µg SEAP-reference vector were transfected into 2.5 × 10^5^ cells using NEPA21 (Nepa Gene). The electroporation conditions were as follows: poration pulse (pulse voltage, 175 V; pulse width, 4 ms; pulse interval, 50 ms; pulse number, 6; voltage decay, 10%; voltage polarity, +) and transfer pulses (pulse voltage, 20 V; pulse width, 50 ms; pulse interval, 50 ms; pulse number, 5 for each polarity; voltage decay, 40%; voltage polarity, +/−). To assess transfection efficiency, SEAP reference vectors were used. The promoter for the reference vector  in each experiment was as follows: *PvGapdh* promoter in Fig. 1a, c, d; *Bmhsp90* promoter in Figs. 1b and 2; OpIE2 promoter in Fig. 3.

### Establishment of stable AcGFP1- or Nluc-expressing cells

SaPe-4, S2 and Sf9 cells were transfected with the vectors containing AcGFP1- and ZeoR-expression cassettes using FuGENE 6 (Promega). Five days after transfection, the SaPe-4 and Sf9 cells were treated with 100 µg/ml zeocin, while S2 cells were treated with 400 µg/ml zeocin. After three weeks of selection in zeocin-containing medium, the cells were subjected to flow cytometry analysis and image acquisition by a CytoFLEX S (Beckman Coulter Life Sciences, Indianapolis, IN, USA) and a BZ-X700 microscope (Keyence, Osaka, Japan), respectively. Similarly, stable Nluc-expressing S2 cells were established. Nluc activity was measured using an ARVO luminometer (PerkinElmer) with the Nano-Glo® Luciferase assay system (Promega).

### Flow cytometry analysis

Transformed cells were stained with DAPI (Dojindo, Kamimashiki, Kumamoto, Japan). A CytoFLEX S flow cytometer (Beckman Coulter), equipped with 375-nm and 488-nm lasers, was used to detect the fluorescence of DAPI and AcGFP1, respectively. The gating hierarchy and dot-plot images of the SaPe-4 cells are shown in Fig. S6. The same gating method was applied to transformed S2 and Sf9 cells.

### Statistical analysis

All data were expressed as mean ± SD. Statistical significance between two groups was examined by the Student *t*-test in Figs 1b,c and 2. Statistical significance among more than two groups was examined by ANOVA followed by Tukey test as a post-hoc test  (Figs 1a, 3, 4, 5, S1 and S7). A *p*-value < 0.05 denoted a statistically significant difference. GraphPad Prism 8 software (GraphPad, San Diego, CA, USA) was used for the statistical analyses.

## Supplementary information

SUPPLEMENTARY INFORMATION
