# Supplementary material for: Identification of a novel strong promoter from the anhydrobiotic midge, Polypedilum vanderplanki, with conserved function in various insect cell lines
Source: Sci Rep. 2019 May 7;9:7004. doi: 10.1038/s41598-019-43441-x (PMC6504868; doi:10.1038/s41598-019-43441-x)
Supplement: Supplementary file 1 — SUPPLEMENTARY INFORMATION [file 41598_2019_43441_MOESM1_ESM.pdf]

Identification of a novel strong promoter from the anhydrobiotic midge, *Polypedilum vanderplanki*, with conserved function in various insect cell lines

Yugo Miyata[1,2,$], Shoko Tokumoto[3,$], Yoichiro Sogame[4,$], Ruslan Deviatiiarov[5], Jun Okada[2], Richard Cornette[2], Oleg Gusev[5,6,7], Elena Shagimardanova[5], Minoru Sakurai[1], Takahiro Kikawada[2,3,*]

[1]Center for Biological Resources and Informatics, Tokyo Institute of Technology, Yokohama, Japan

[2]Anhydrobiosis Research Group, Molecular Biomimetics Research Unit, Institute of Agrobiological Sciences, National Institute of Agriculture and Food Research Organization (NARO), Tsukuba, Japan

[3]Department of Integrated Biosciences, Graduate School of Frontier Sciences, The University of Tokyo, Kashiwa, Japan

[4]Department of Applied Chemistry and Biochemistry, National Institute of Technology, Fukushima College, Iwaki, Japan

[5]Institute of Fundamental Medicine and Biology, Kazan Federal University, Kazan, Russian Federation

[6]RIKEN-KFU Translational Genomics Unit, RIKEN Cluster for Science, Technology and Innovation Hub, RIKEN, Yokohama, Japan

[7]Preventive Medicine and Diagnosis Innovation Program, RIKEN Cluster for Science, Technology and Innovation Hub, RIKEN, Yokohama, Japan

[$]These authors contributed equally to this study.

**Corresponding author:*

Takahiro Kikawada, PhD

Anhydrobiosis Research Group, Molecular Biomimetics Research Unit, Institute of Agrobiological Sciences, National Institute of Agriculture and Food Research Organization, Ohwashi 1-2, Tsukuba, 305-8634, Japan

Tel: +81 298386170, Fax: +81 298386170

Email: kikawada@affrc.go.jp

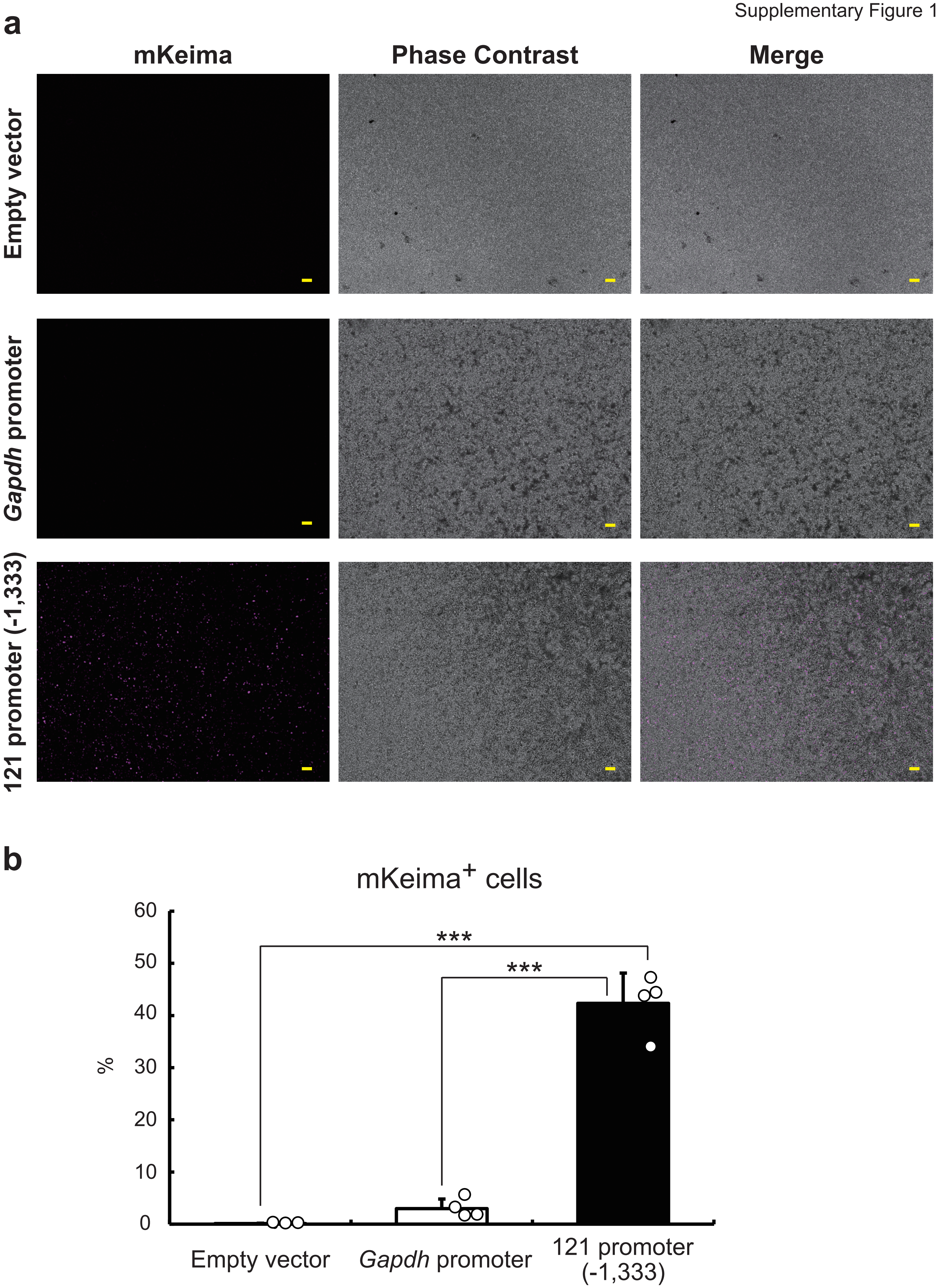
a
mKeima
Phase Contrast
Merge
Empty vector
Gapdh promoter
121 promoter (-1,333)
b
mKeima+ cells
60
50
40
30
20
10
0
%
***
***
Empty vector
Gapdh promoter
121 promoter
(-1,333)

Figure S1

Comparison of the promoter activities of the *PvGapdh* and 121 promoters.

The *PvGapdh* and 121 promoters were ligated to the mKeima gene, and the plasmid vectors were transfected into Pv11 cells. Images were acquired three days after transfection using a BZ-X700 fluorescence microscope. Merged images are shown on the right (a). The proportions of $mKeima^+$ cells in the live cell population (%) were analyzed using a CytoFLEX S flow cytometer (b). Scale bars, 50 μm. The values are expressed as mean ± SD. *** $p < 0.001$; n=3-4 in each group.

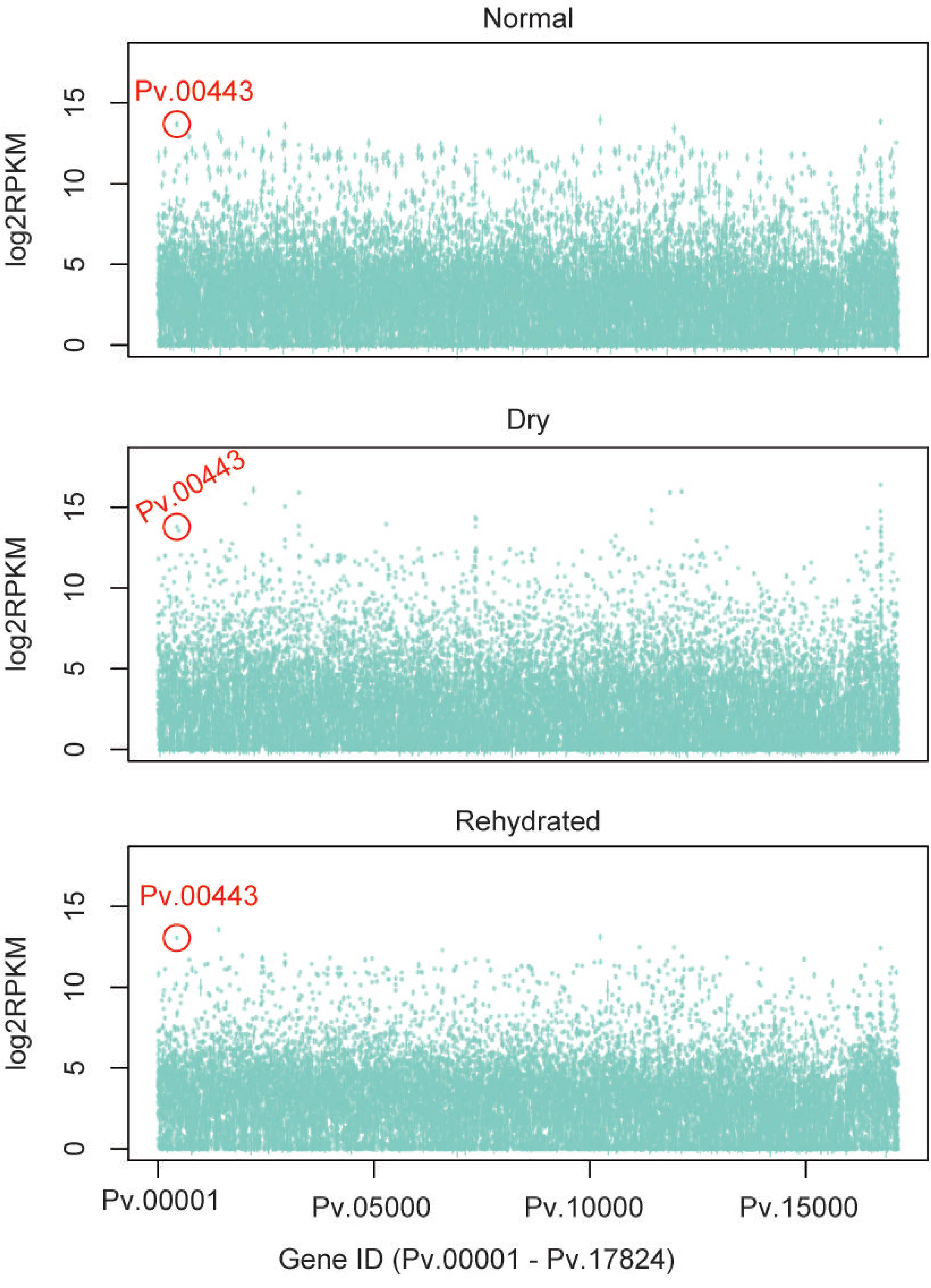
Normal
Pv.00443
log2RPKM
0
5
10
15
Dry
Pv.00443
log2RPKM
0
5
10
15
Rehydrated
Pv.00443
log2RPKM
0
5
10
15
Pv.00001
Pv.05000
Pv.10000
Pv.15000
Gene ID (Pv.00001 - Pv.17824)

Figure S2

Global search for constitutively highly expressed genes in *P. vanderplanki* larvae.

Expression levels (log2RPKM) of all *P. vanderplanki* genes from MidgeBase (http://bertone.nises-f.affrc.go.jp/midgebase/) are shown. The *Pv.00443* gene is consistently highly expressed during normal, dry and rehydrated conditions. "Dry" means 48 h after desiccation and "Rehydrated" means 24 h after rehydration.

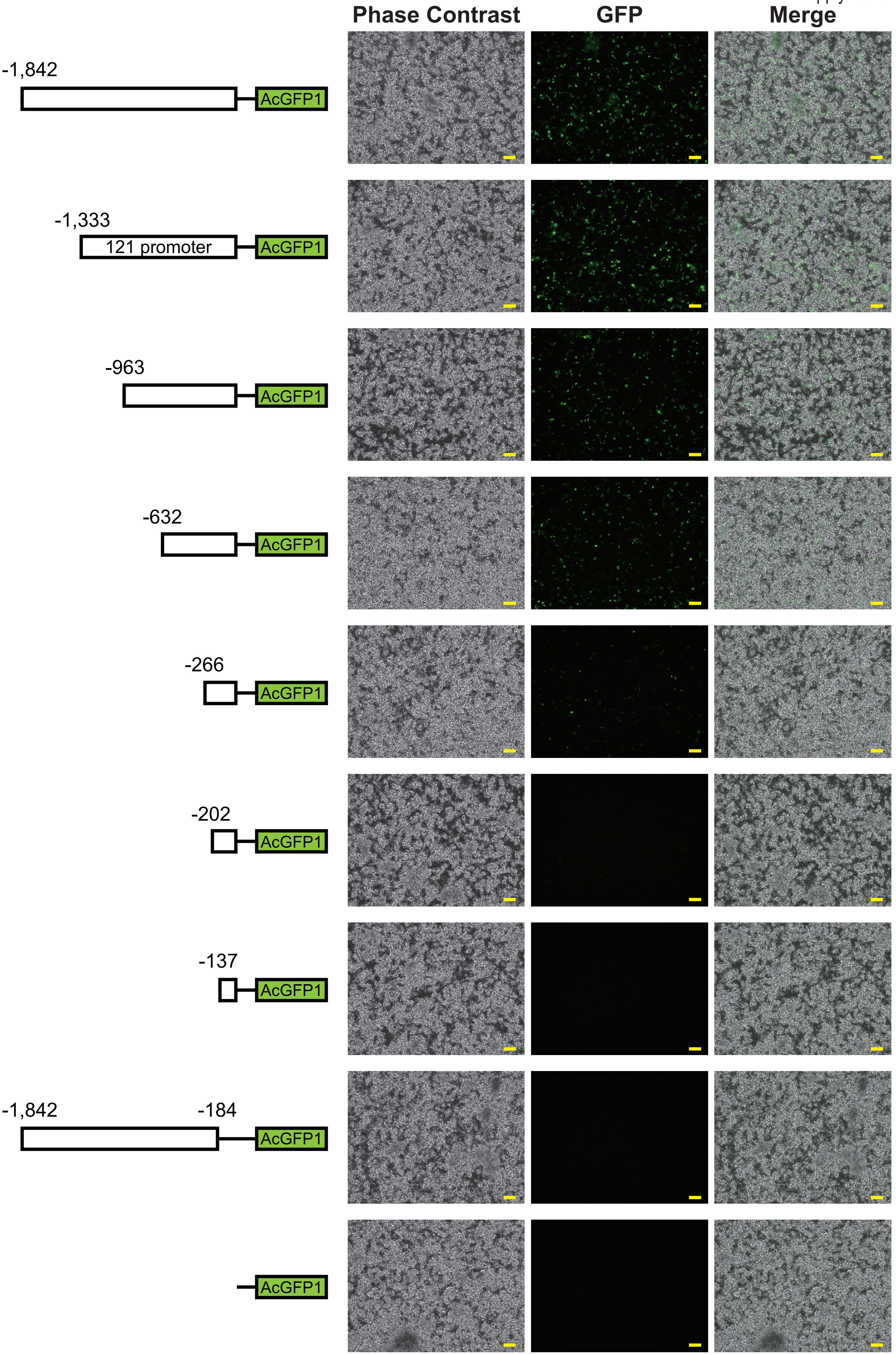
Phase Contrast
GFP
Merge
-1,842
AcGFP1
-1,333
121 promoter
AcGFP1
-963
AcGFP1
-632
AcGFP1
-266
AcGFP1
-202
AcGFP1
-137
AcGFP1
-1,842
-184
AcGFP1
AcGFP1

Figure S3

AcGFP1 expression under the control of deletion mutants of the 121 promoter.

The constructs for AcGFP1 gene expression under the control of a series of deletion mutants of the 121 promoter are shown on the left. Images were obtained three days after transfection and show phase contrast (left) and GFP fluorescence (middle). The merged images are shown on the right. Scale bars, 100 µm.

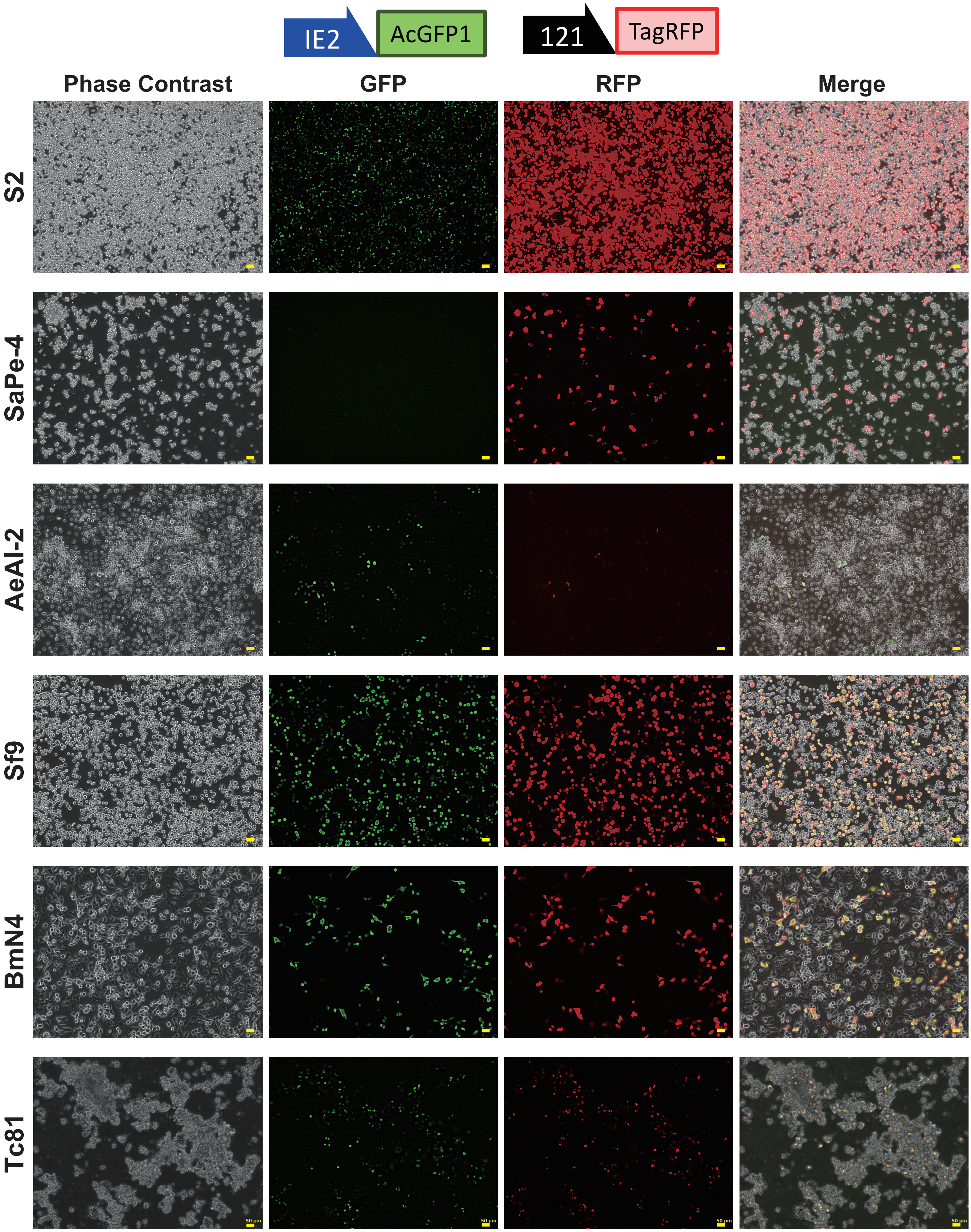
IE2
AcGFP1
121
TagRFP
Phase Contrast
GFP
RFP
Merge
S2
SaPe-4
AeAl-2
Sf9
BmN4
Tc81

Figure S4

Comparison of OpIE2 and 121 promoter activities in various insect cell lines.

The OpIE2 and 121 promoters were ligated to the AcGFP1 and TagRFP genes, respectively, and the plasmid vectors were transfected into various insect cell lines as indicated on the left. The images were acquired three days after transfection, and the merged images are shown on the right. Scale bars, 50 μm.

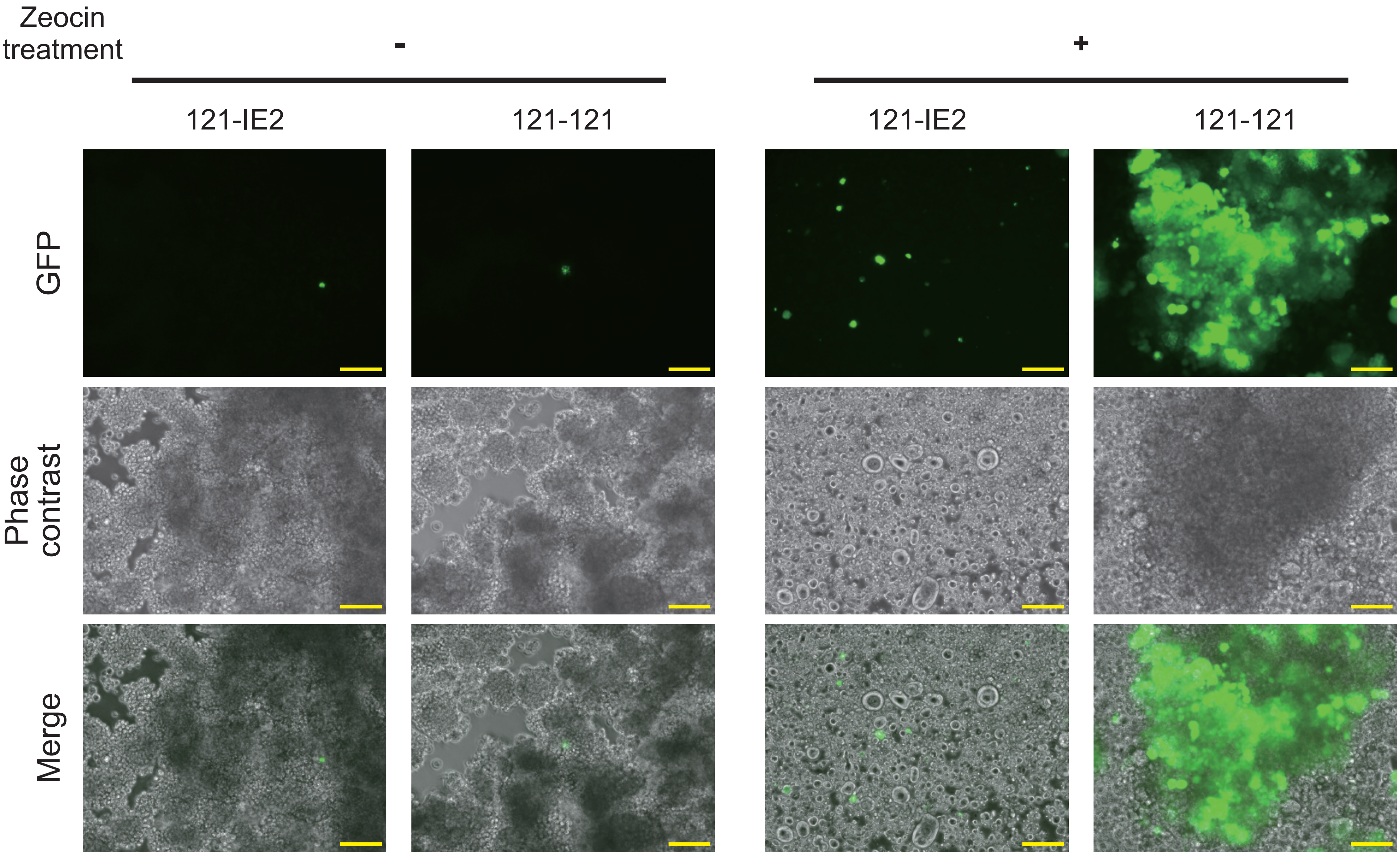
Zeocin treatment
-
+
121-IE2
121-121
121-IE2
121-121
GFP
Phase contrast
Merge

Figure S5

Magnified images of Figure 4b. Scale bars, 100 μm.

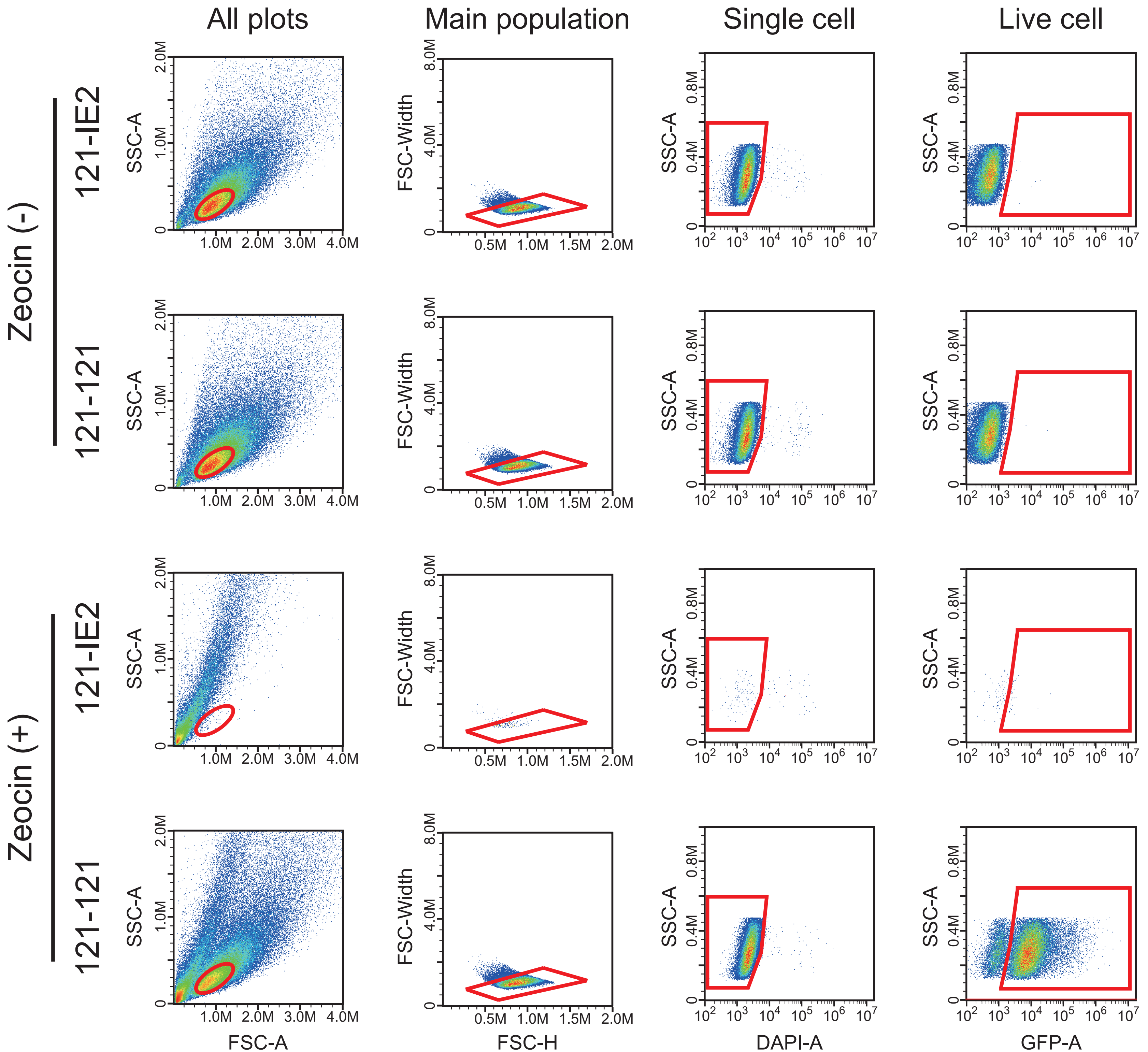
All plots
Main population
Single cell
Live cell
Zeocin (-)
Zeocin (+)
121-IE2
121-121
SSC-A
FSC-Width
FSC-A
FSC-H
DAPI-A
GFP-A

Figure S6

Gating hierarchy and dot-plot images of transformed SaPe-4 cells in flow cytometry experiments.

Representative dot-plot images of the experimental groups are shown, and all gates are colored red. The gating hierarchy is main population (left panels) - single cell (doublet discrimination, left middle panels) - live cell (right middle panels) - GFP^{+} cell (right panels). Most 121-IE2-transfected cells died after zeocin selection (middle lower panels), while most live cells were AcGFP1-positive in the 121-121-transfected group (lower panels).

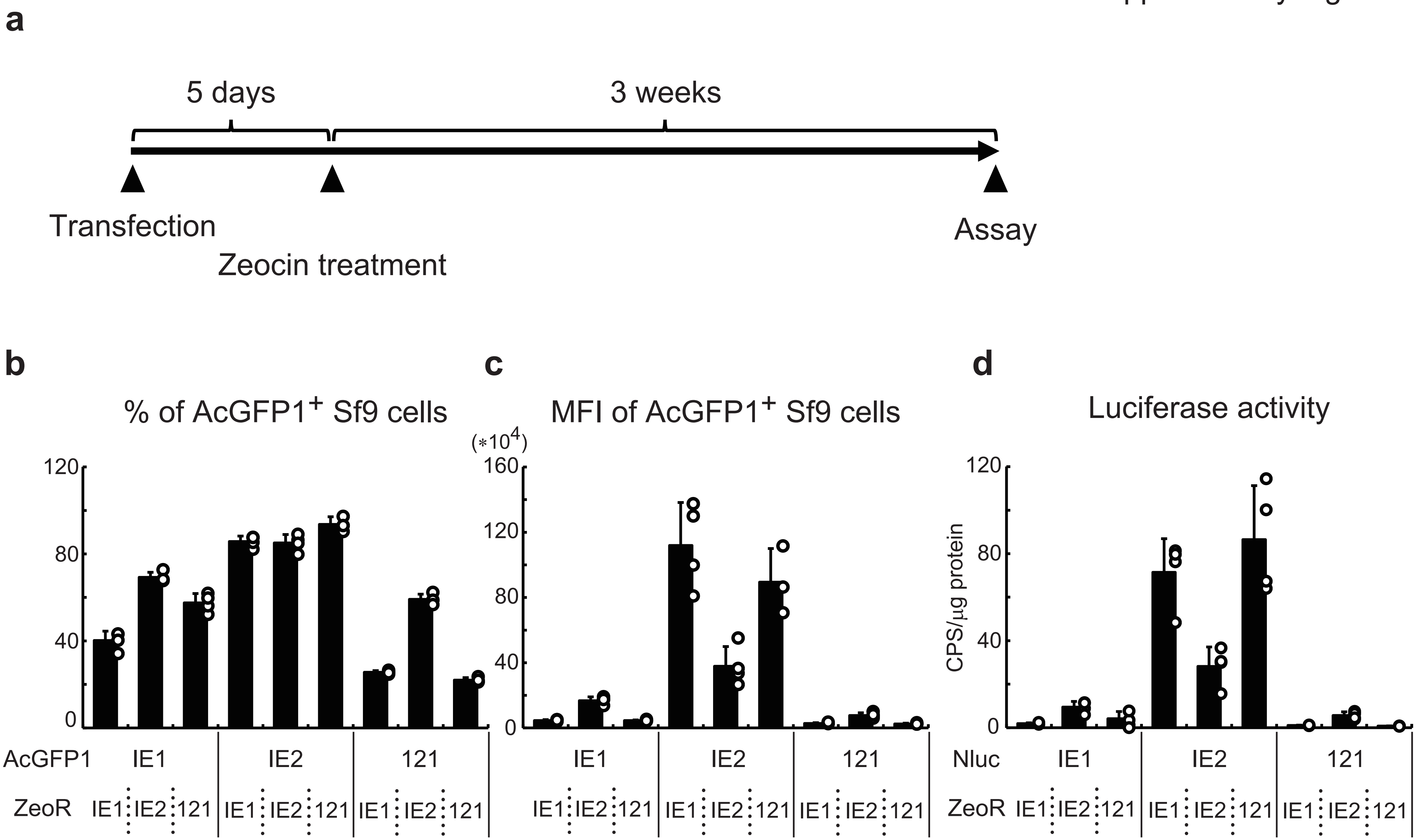
a
5 days
3 weeks
Transfection
Zeocin treatment
Assay
b
% of AcGFP1+ Sf9 cells
120
80
40
0
AcGFP1
IE1
IE2
121
ZeoR
IE1 IE2 121 IE1 IE2 121 IE1 IE2 121
c
MFI of AcGFP1+ Sf9 cells
(*10^4)
160
120
80
40
0
IE1
IE2
121
IE1 IE2 121 IE1 IE2 121 IE1 IE2 121
d
Luciferase activity
CPS/μg protein
120
80
40
0
Nluc
IE1
IE2
121
ZeoR
IE1 IE2 121 IE1 IE2 121 IE1 IE2 121

Figure S7

Use of the 121 promoter for the establishment of stably transfected Sf9 cells.

The experimental scheme is shown in (a). Nine plasmid vectors for the expression of AcGFP1/Nluc and ZeoR were transfected into Sf9 cells. After zeocin selection, the cells were assessed for AcGFP1 fluorescence or luciferase activity. In the AcGFP1-expressing cells, the proportions and MFIs of GFP^+ cells in the live cell population were analyzed by a CytoFLEX S flow cytometer (b and c). In the Nluc-expressing cells, the luciferase activities were measured (d). The values are expressed as mean ± SD; n=3-4 in each group. Statistical analysis is presented on Tables S1-S4.

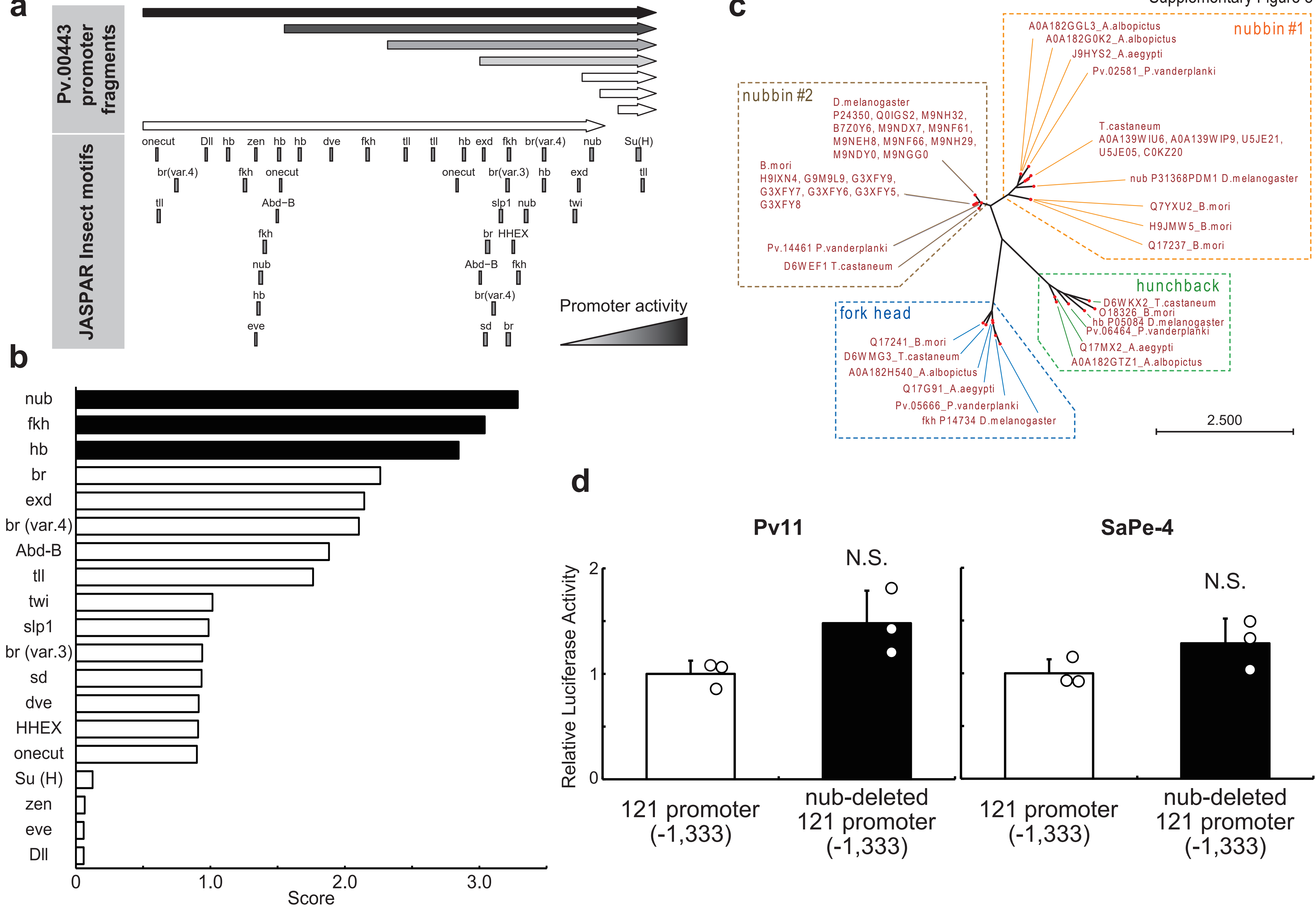
a
Pv.00443 promoter fragments
JASPAR Insect motifs
Promoter activity
b
nub
fkh
hb
br
exd
br (var.4)
Abd-B
tll
twi
slp1
br (var.3)
sd
dve
HHEX
onecut
Su (H)
zen
eve
Dll
Score
c
nubbin #1
nubbin #2
hunchback
fork head
2.500
d
Pv11
SaPe-4
N.S.
Relative Luciferase Activity
121 promoter (-1,333)
nub-deleted 121 promoter (-1,333)

Figure S8

Analysis of known motif sequences in the 121 promoter.

Known insect TFBS motifs in the 121 promoter sequence were annotated (a). The impact of predicted TFBSs on 121 promoter activity was calculated as follows: Score= $\sum_{i}^{L} Act(i) * I$, where L - number of fragment, I - motif similarity score, Act - difference of activity between neighbor sequences normalized to the length of removed fragment (b). Phylogenetic comparison of the top three transcription factors for *D. melanogaster*, *B. mori*, *T. castaneum*, *P. vanderplanki*, *A. albopictus* and *Aedes aegypti* revealed two distinct types of nubbin molecule for these insects (c). The relative activities of the 121 promoters with or without the nubbin #2 binding motif were measured in Pv11 and Sape-4 cells (d). Normalized values are expressed as mean ± SD; N.S., not significant; n=3 in each group.

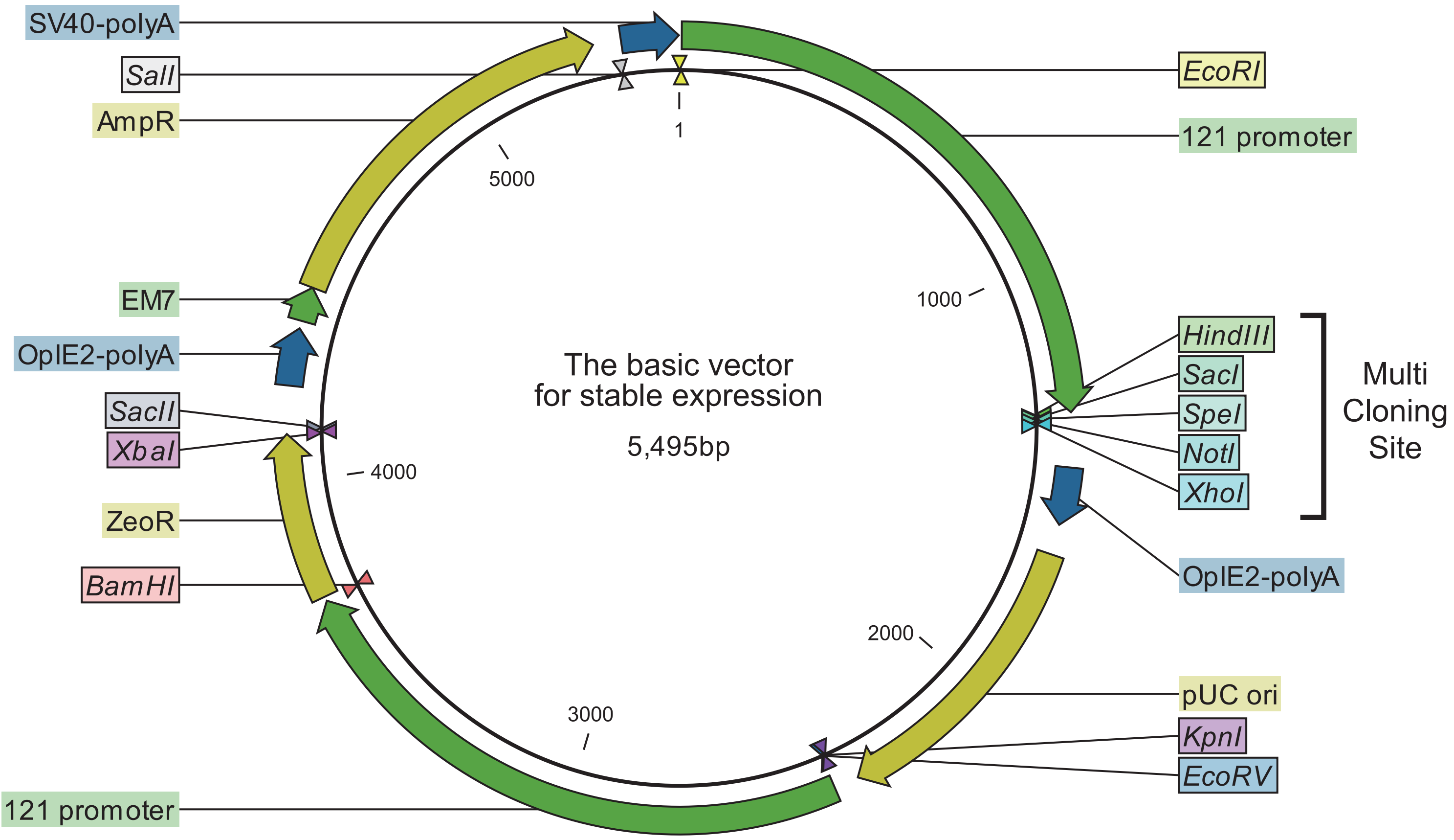
SV40-polyA
SalI
AmpR
EM7
OpIE2-polyA
SacII
XbaI
ZeoR
BamHI
121 promoter
EcoRI
121 promoter
HindIII
SacI
SpeI
NotI
XhoI
Multi Cloning Site
OpIE2-polyA
pUC ori
KpnI
EcoRV
1
1000
2000
3000
4000
5000
The basic vector for stable expression
5,495bp

Figure S9

The map of the basic vector for stable expression.

The multi-cloning site of the vector includes *Hin*dIII, *Sac*I, *Spe*I, *Not*I and *Xho*I sites.

Supplementary Figure10

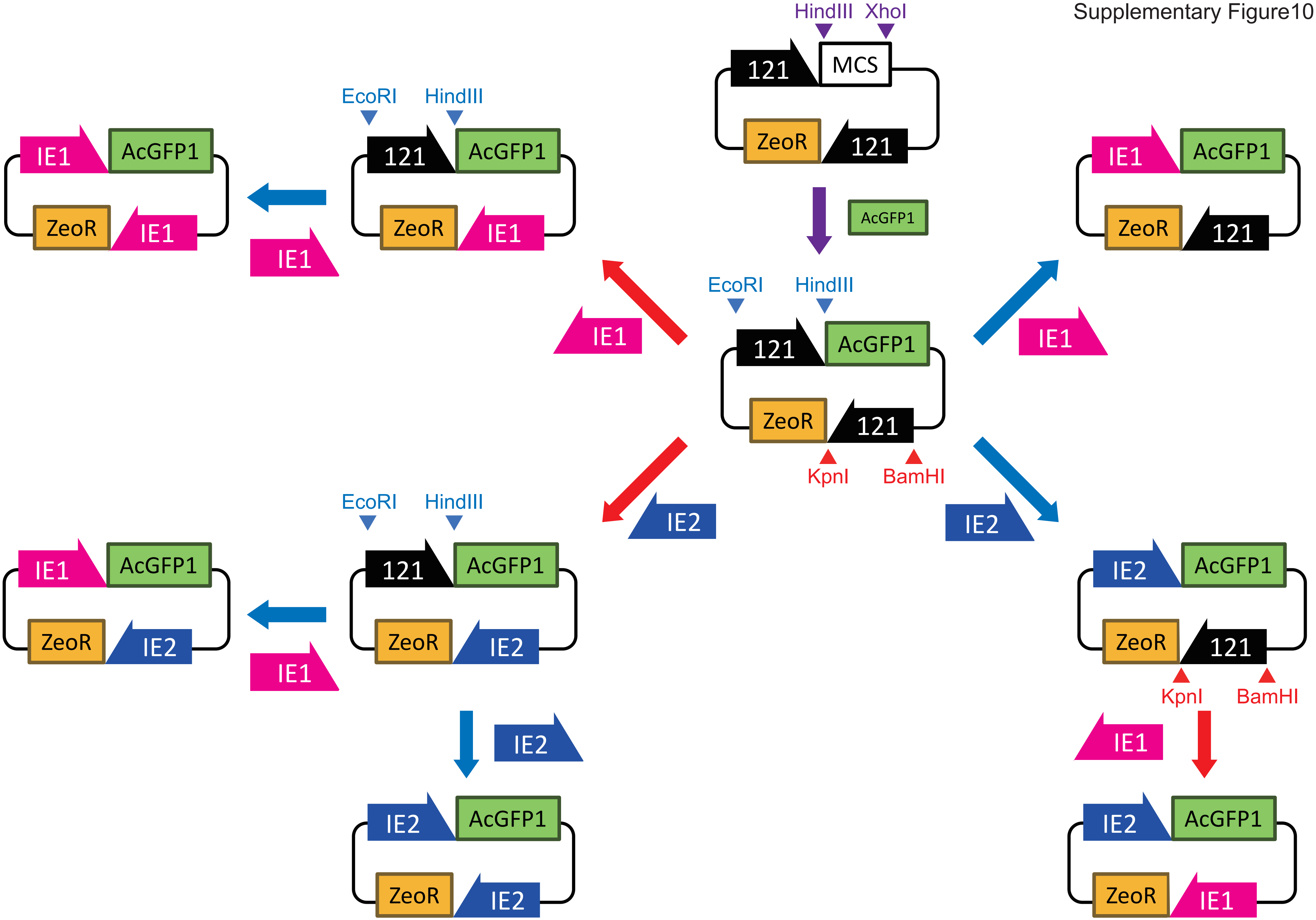

Figure S10

Vector construction scheme for stable expression of AcGFP1.

The basic vector was digested with *Hin*dIII and *Xho*I, and a PCR fragment of the AcGFP1 gene was ligated by HiFi assembly. Then, the vector was digested with *Eco*RI and *Hin*dIII, or *Kpn*I and *Bam*HI, and a PCR fragment of the OpIE1 or OpIE2 promoter was ligated by HiFi assembly.

Supplementary Table 1: Two-way ANOVA in Figures S7b-d.

Cell	Data Source (Dependent Variable)	Source of Variation	% of total variation	*p*-value
Sf9	Figure S7b (% of AcGFP1+ cells)	Interaction	12.01	<0.0001
		AcGFP1	74.94	<0.0001
		ZeoR	12.43	<0.0001
	Figure S7c (MFI of AcGFP1+ cells)	Interaction	17.72	<0.0001
		AcGFP1	72.93	<0.0001
		ZeoR	3.997	0.0016
	Figure S7d (Luciferase activity)	Interaction	15.73	<0.0001
		Nluc	72.02	<0.0001
		ZeoR	4.097	0.0039

Supplementary Table 2: Statistical analysis of percentages of AcGFP1⁺ Sf9 cells in Figure S7b.

	IE1-IE1	IE1-IE2	IE1-121	IE2-IE1	IE2-IE2	IE2-121	121-IE1	121-IE2	121-121
IE1-IE1		***	***	***	***	***	***	***	***
IE1-IE2	-		***	***	***	***	***	**	***
IE1-121	-	-		***	***	***	***	ns	***
IE2-IE1	-	-	-		ns	*	***	***	***
IE2-IE2	-	-	-	-		*	***	***	***
IE2-121	-	-	-	-	-		***	***	***
121-IE1	-	-	-	-	-	-		***	ns
121-IE2	-	-	-	-	-	-	-		***
121-121	-	-	-	-	-	-	-	-	

Statistical analysis was performed by Tukey test as a post-hoc test for two-way ANOVA.

* $p < 0.05$; ** $p < 0.01$; *** $p < 0.001$; ns, not significant.

Supplementary Table 3: Statistical analysis of MFIs of AcGFP1$^+$ Sf9 cells in Figure S7c.

	IE1-IE1	IE1-IE2	IE1-121	IE2-IE1	IE2-IE2	IE2-121	121-IE1	121-IE2	121-121
IE1-IE1		ns	ns	***	**	***	ns	ns	ns
IE1-IE2	-		ns	***	ns	***	ns	ns	ns
IE1-121	-	-		***	**	***	ns	ns	ns
IE2-IE1	-	-	-		***	ns	***	***	***
IE2-IE2	-	-	-	-		***	**	*	**
IE2-121	-	-	-	-	-		***	***	***
121-IE1	-	-	-	-	-	-		ns	ns
121-IE2	-	-	-	-	-	-	-		ns
121-121	-	-	-	-	-	-	-	-	

Statistical analysis was performed by Tukey test as a post-hoc test for two-way ANOVA.

* $p < 0.05$; ** $p < 0.01$; *** $p < 0.001$; ns, not significant.

Supplementary Table 4: Statistical analysis of luciferase activities of AcGFP1$^+$ Sf9 cells in Figure S7d.

	IE1-IE1	IE1-IE2	IE1-121	IE2-IE1	IE2-IE2	IE2-121	121-IE1	121-IE2	121-121
IE1-IE1		ns	ns	***	*	***	ns	ns	ns
IE1-IE2	-		ns	***	ns	***	ns	ns	ns
IE1-121	-	-		***	ns	***	ns	ns	ns
IE2-IE1	-	-	-		***	ns	***	***	***
IE2-IE2	-	-	-	-		***	*	ns	*
IE2-121	-	-	-	-	-		***	***	***
121-IE1	-	-	-	-	-	-		ns	ns
121-IE2	-	-	-	-	-	-	-		ns
121-121	-	-	-	-	-	-	-	-	

Statistical analysis was performed by Tukey test as a post-hoc test for two-way ANOVA.

* $p < 0.05$; ** $p < 0.01$; *** $p < 0.001$; ns, not significant.

Supplementary Table 5: Predicted contribution of nubbin binding sites on the 121 promoter activity in Figure S8.

motif number of JASPR	motif ID	input sequence	start	stop	strand	score	*p*-value	*q*-value	matched sequence
MA0197.2	nub	121 promoter	461	472	-	16.2418	1.55E-06	0.00329	TATGCAAATTAA
MA0197.2	nub	121 promoter	226	237	+	11.2418	5.69E-05	0.06	TATTGAAATGAG
MA0197.2	nub	121 promoter	1413	1424	+	10.4725	8.45E-05	0.06	AATTTAAATGAG

The most probable nubbin binding site was listed on the top of the table, and its impact on the 121 promoter activity was examined in Pv11 and SaPe-4 cells (Fig. S8d).

Supplementary Table 6: Primers for the construction of the basic vector for stable expression in Figure S9.

PCR fragment	forward primer (5'-3')	reverse primer (5'-3')
AmpR	CAAGCGCGTGCGAATTAATTCGGATCTCTG-CAGCACGTGTTGACAATTAATCATCGGCAT-AGTATATCGGCATAGTATAATACGACTCAC-TATAGGAGGGCCACCATGAGTATTCAACAT-TTCCG	TCGGCGTCGGTTACCAATGCTTAATCAGTG
121 promoter for GOI	GCATTGGTAACCGACGCCGACCAACACC	CTAGTGAGCTCAAGCTTTTTTTTTCAGAAAA-TATTTTCTTTTGTCCAACGATGTTATTCGT-TTAG
121 promoter for ZeoR	GGGATTTTGGGGTACCGATATCCTTCAATT-ATGATACATGAATAAACAAAATATTAAAG	GGCCATGTTGGGATCCTTTTTTCAGAAAAT-ATTTTCTTTTGTC
pUC ori	TCTGAAAAAAAAAGCTTGAGCTCACTAGTGC-GGCCGCTCGAGTTCGAAGGTAAGCCTATC	TGAAGGATATCGGTACCCCAAAATCCCTTA-ACGTG
ZeoR	TCTGAAAAAAGGATCCCAACATGGCCAAGT-TGACCAGTG	CGCGGGCCCTCTAGATTAGTCCTGCTCCTC-GGC
poly(A) signal for ZeoR	GCAGGACTAATCTAGAGGGCCCGCGGTTCG-AAGGTAAGCCTATCCC	GAGATCCGAATTAATTCGCACGCGCTTGAA-AGGAGTG

The primers were designed using the NEBuilder Assembly Tool Version1 (https://nebuilderv1.neb.com/).

Supplementary Data 1

The 1,842 base sequence in Figure 1.

The region of the 121 promoter was shown in red. The highlighted region shows a putative nubbin binding motif (related to Fig. S8).

TCGTGAGTCAATTTAAAAATTACTTACTAATATTTGATAATTAATCTTGACTTTT

TCTTCTTTTTTCTCACAAGTGAATTGAATATGAAAGAAAATCGAGAAGATCTA

TCTCTTTTTGGAGTTCGAAGCTGCAGTTGCATCGCATGCGTTCAGAATTTATC

TTTAAGTCACATAAATGATAAAAATATGATGCAAATGGCTAGAGTGTACAATT

TTCATTTTGATACTATTGAAATGAGAAATATTTCGCATGCAATGGAAATAATTT

CAACACTATTTGACATTCTCAACAAGTGCGAAGAACCAATAGTTTTGGAAAT

GCTTTTTTGCTGTAAAATTATCAAGGAAATATTCAACAGTCTCGTCGATCGCTT

AATGTTTCCGATATAAAATTTAAAATTTTTTACTGTGTAACATCTTATCTATTCC

ATATTTTTTTGATTCATTTTCTCAACCACATATTTAATTTGCATAATTGTTATTAA

ACTTTTGTTTAACTACTAAAAAAACTTCAATTATGATACATGAATAAACAAAA

TATTAAAGAATAAAATTGTTTTTCTTCATTCATATTGAATTTTTATTTATTATTTT

GTTAACTTTTGTCTTTTAAAAATTTCAAAGATATTTCATAAATTCATCTGAAAT

TCTCAGATTTATAATGTAATGTTGAAAAAATCGTATTTATTTTTTTGCGTAAATG

AAAGGGAAATTGATTTCGCAATCCAAAAGAAGAATTTCTTTTACATTTTGTAT

TTAAAGATACGCAAAAAAGAACACAAATTCATAATAATTTTCAAAGTCAACA

GAAGTACATTGCTTCTATAAAAATTATTTAAAATTTAAAATTTGTGAAAAGAAT

TTTAAAATGTGTGGTAGAAATAAAAATCTTAATAAAAGTCAGTGCGAGTGTCA

GCATCATCATCATCATCATTTTTATATCAATGAACAGTTAATAGAACCTGAGGA

AAAATCATCAAAATTCAAATGATTCAGCAAACCATCAATACATCACTATTTTTA

CTTCAACGATAAGCAAATAAATAAAGAAGATTGTCAGCCGAATGTTGCTACA

CTCGCGAAAAATACAGTAAAAGAACATTTCATTCAACATCAAAAAGCTGCAT

CTCAAGAAAAAAATTCAGGCTGCTATCACTTTGAACATAATCCAATTGATAAG

AAATGGGCACAGTATTTTGAAGAGAGATTTGGAAAATCACATGGACATCACC

ATTATCATTCGTTCAATAATGATGATTTTCCAAAAATGTTTTTCAACAAAAAAT

ATTTTGAAGGAAGTTTCCTTGATTAAGCAATTCATATATTTGAAAGAGAATTTT

TGCATTTGATTTTTTTTTCATAAAAATTAATTTGTCGTTTATAAAACATATTTGT

CAGTATGTTTATTTTAAAAAATTTAAATGAGTAAAAAAACTAATGAAAACTAT

AAATAAGTGAATTTTTATTTCATTTGTACAAACAATGATTATCATCATTATCTCA

TCAATTTCATCACTTTTAGAGAATTTTCACAAAAAAAAATTATTTTTCTACAGA

AAAATCACAAAATAATGAAATTCTTATCAATATAAAAAATGCATGTCATTACCG

GCAATTACTCATATACAAAACAAACAAGCACAAACACGTTCAAAATCATATTT

TCCTCACTATACAATATAATTATTGTACACATGCTCATTTATTTCACAAGAATTG

TAAAGAATATTGTGAGTGTATGTGAAGCATATAAAAAGCAGATTAAATCGTAC

AACATTCAGTTGACTTATGATTTCTAAACGAATAACATCGTTGGACAAAAGAA

AATATTTTCTGAAAAAA

Supplementary Data 2

Full length sequence of the basic vector for stable expression related to Figure S9.

The region of 121 promoter is shown in red.

GAATTCCTTCAATTATGATACATGAATAAACAAAATATTAAAGAATAAAATTGT

TTTTCTTCATTCATATTGAATTTTTATTTATTATTTTGTTAACTTTTGTCTTTTAA

AAATTTCAAAGATATTTCATAAATTCATCTGAAATTCTCAGATTTATAATGTAAT

GTTGAAAAAATCGTATTTATTTTTTTGCGTAAATGAAAGGGAAATTGATTTCG

CAATCCAAAAGAAGAATTTCTTTTACATTTTGTATTTAAAGATACGCAAAAAA

GAACACAAATTCATAATAATTTTCAAAGTCAACAGAAGTACATTGCTTCTATA

AAAATTATTTAAAATTTAAAATTTGTGAAAAGAATTTTAAAATGTGTGGTAGA

AATAAAAATCTTAATAAAAGTCAGTGCGAGTGTCAGCATCATCATCATCATCA

TTTTTATATCAATGAACAGTTAATAGAACCTGAGGAAAAATCATCAAAATTCA

AATGATTCAGCAAACCATCAATACATCACTATTTTTACTTCAACGATAAGCAA

ATAAATAAAGAAGATTGTCAGCCGAATGTTGCTACACTCGCGAAAAATACAG

TAAAAGAACATTTCATTCAACATCAAAAAGCTGCATCTCAAGAAAAAAATTC

AGGCTGCTATCACTTTGAACATAATCCAATTGATAAGAAATGGGCACAGTATT

TTGAAGAGAGATTTGGAAAATCACATGGACATCACCATTATCATTCGTTCAAT

AATGATGATTTTCCAAAAATGTTTTTCAACAAAAAATATTTTGAAGGAAGTTT

CCTTGATTAAGCAATTCATATATTTGAAAGAGAATTTTTGCATTTGATTTTTTTT

TCATAAAAATTAATTTGTCGTTTATAAAACATATTTGTCAGTATGTTTATTTTAA

AAAATTTAAATGAGTAAAAAAACTAATGAAAACTATAAATAAGTGAATTTTTA

TTTCATTTGTACAAACAATGATTATCATCATTATCTCATCAATTTCATCACTTTT

AGAGAATTTTCACAAAAAAAAATTATTTTTCTACAGAAAAATCACAAAATAAT

GAAATTCTTATCAATATAAAAAATGCATGTCATTACCGGCAATTACTCATATAC

AAAACAAACAAGCACAAACACGTTCAAAATCATATTTTCCTCACTATACAATA

TAATTATTGTACACATGCTCATTTATTTCACAAGAATTGTAAAGAATATTGTGA

GTGTATGTGAAGCATATAAAAAGCAGATTAAATCGTACAACATTCAGTTGACT

TATGATTTCTAAACGAATAACATCGTTGGACAAAAGAAAATATTTTCTGAAAA

AAAAGCTTGAGCTCACTAGTGCGGCCGCTCGAGTTCGAAGGTAAGCCTATCC

CTAACCCTCTCCTCGGTCTCGATTCTACGCGTACCGGTCATCATCACCATCAC

CATTGAGTTTATCTGACTAAATCTTAGTTTGTATTGTCATGTTTTAATACAATAT

GTTATGTTTAAATATGTTTTTAATAAATTTTATAAAATAATTTCAACTTTTATTGT

AACAACATTGTCCATTTACACACTCCTTTCAAGCGCGTGGGATCGATGCTCAC

TCAAAGGCGGTAATACGGTTATCCACAGAATCAGGGGATAACGCAGGAAAGA

ACATGTGAGCAAAAGGCCAGCAAAAGGCCAGGAACCGTAAAAAGGCCGCG

TTGCTGGCGTTTTTCCATAGGCTCCGCCCCCCTGACGAGCATCACAAAAATCG

ACGCTCAAGTCAGAGGTGGCGAAACCCGACAGGACTATAAAGATACCAGGC

GTTTCCCCCTGGAAGCTCCCTCGTGCGCTCTCCTGTTCCGACCCTGCCGCTTA

CCGGATACCTGTCCGCCTTTCTCCCTTCGGGAAGCGTGGCGCTTTCTCATAGC

TCACGCTGTAGGTATCTCAGTTCGGTGTAGGTCGTTCGCTCCAAGCTGGGCTG

TGTGCACGAACCCCCCGTTCAGCCCGACCGCTGCGCCTTATCCGGTAACTATC

GTCTTGAGTCCAACCCGGTAAGACACGACTTATCGCCACTGGCAGCAGCCAC

TGGTAACAGGATTAGCAGAGCGAGGTATGTAGGCGGTGCTACAGAGTTCTTG

AAGTGGTGGCCTAACTACGGCTACACTAGAAGAACAGTATTTGGTATCTGCG

CTCTGCTGAAGCCAGTTACCTTCGGAAAAAGAGTTGGTAGCTCTTGATCCGG

CAAACAAACCACCGCTGGTAGCGGTGGTTTTTTTGTTTGCAAGCAGCAGATT

ACGCGCAGAAAAAAAGGATCTCAAGAAGATCCTTTGATCTTTTCTACGGGGT

CTGACGCTCAGTGGAACGAAAACTCACGTTAAGGGATTTTGGGGTACCGATA

TCCTTCAATTATGATACATGAATAAACAAAATATTAAAGAATAAAATTGTTTTT

CTTCATTCATATTGAATTTTTATTTATTATTTTGTTAACTTTTGTCTTTTAAAAAT

TTCAAAGATATTTCATAAATTCATCTGAAATTCTCAGATTTATAATGTAATGTTG

AAAAAATCGTATTTATTTTTTTGCGTAAATGAAAGGGAAATTGATTTCGCAAT

CCAAAAGAAGAATTTCTTTTACATTTTGTATTTAAAGATACGCAAAAAAGAAC

ACAAATTCATAATAATTTTCAAAGTCAACAGAAGTACATTGCTTCTATAAAAA

TTATTTAAAATTTAAAATTTGTGAAAAGAATTTTAAAATGTGTGGTAGAAATA

AAAATCTTAATAAAAGTCAGTGCGAGTGTCAGCATCATCATCATCATCATTTTT

ATATCAATGAACAGTTAATAGAACCTGAGGAAAAATCATCAAAATTCAAATGA

TTCAGCAAACCATCAATACATCACTATTTTTACTTCAACGATAAGCAAATAAAT

AAAGAAGATTGTCAGCCGAATGTTGCTACACTCGCGAAAAATACAGTAAAAG

AACATTTCATTCAACATCAAAAAGCTGCATCTCAAGAAAAAAATTCAGGCTG

CTATCACTTTGAACATAATCCAATTGATAAGAAATGGGCACAGTATTTTGAAG

AGAGATTTGGAAAATCACATGGACATCACCATTATCATTCGTTCAATAATGAT

GATTTTCCAAAAATGTTTTTCAACAAAAAATATTTTGAAGGAAGTTTCCTTGA

TTAAGCAATTCATATATTTGAAAGAGAATTTTTGCATTTGATTTTTTTTTCATAA

AAATTAATTTGTCGTTTATAAAACATATTTGTCAGTATGTTTATTTTAAAAAATT

TAAATGAGTAAAAAAACTAATGAAAACTATAAATAAGTGAATTTTTATTTCATT
TGTACAAACAATGATTATCATCATTATCTCATCAATTTCATCACTTTTAGAGAAT
TTTCACAAAAAAAAATTATTTTTCTACAGAAAAATCACAAAATAATGAAATTC
TTATCAATATAAAAAATGCATGTCATTACCGGCAATTACTCATATACAAAACAA
ACAAGCACAAACACGTTCAAAATCATATTTTCCTCACTATACAATATAATTATT
GTACACATGCTCATTTATTTCACAAGAATTGTAAAGAATATTGTGAGTGTATGT
GAAGCATATAAAAAGCAGATTAAATCGTACAACATTCAGTTGACTTATGATTT
CTAAACGAATAACATCGTTGGACAAAAGAAAATATTTTCTGAAAAAAggatccca
acATGGCCAAGTTGACCAGTGCCGTTCCGGTGCTCACCGCGCGCGACGTCGC
CGGAGCGGTCGAGTTCTGGACCGACCGGCTCGGGTTCTCCCGGGACTTCGT
GGAGGACGACTTCGCCGGTGTGGTCCGGGACGACGTGACCCTGTTCATCAG
CGCGGTCCAGGACCAGGTGGTGCCGGACAACACCCTGGCCTGGGTGTGGGT
GCGCGGCCTGGACGAGCTGTACGCCGAGTGGTCGGAGGTCGTGTCCACGAA
CTTCCGGGACGCCTCCGGGCCGGCCATGACCGAGATCGGCGAGCAGCCGTG
GGGGCGGGAGTTCGCCCTGCGCGACCCGGCCGGCAACTGCGTGCACTTCGT
GGCCGAGGAGCAGGACTAAtctagaGGGCCCGCGGTTCGAAGGTAAGCCTATCC
CTAACCCTCTCCTCGGTCTCGATTCTACGCGTACCGGTCATCATCACCATCAC
CATTGAGTTTATCTGACTAAATCTTAGTTTGTATTGTCATGTTTTAATACAATAT
GTTATGTTTAAATATGTTTTTAATAAATTTTATAAAATAATTTCAACTTTTATTGT
AACAACATTGTCCATTTACACACTCCTTTCAAGCGCGTGCGAATTAATTCGGA
TCTCTGCAGCACGTGTTGACAATTAATCATCGGCATAGTATATCGGCATAGTAT
AATACGACTCACTATAGGAGGGCCACCATGAGTATTCAACATTTCCGTGTCGC

CCTTATTCCCTTTTTTGCGGCATTTTGCCTTCCTGTTTTTGCTCACCCAGAAAC

GCTGGTGAAAGTAAAAGATGCTGAAGATCAGTTGGGTGCACGAGTGGGTTA

CATCGAACTGGATCTCAACAGCGGTAAGATCCTTGAGAGTTTTCGCCCCGAA

GAACGTTTTCCAATGATGAGCACTTTTAAAGTTCTGCTATGTGGCGCGGTATT

ATCCCGTATTGACGCCGGGCAAGAGCAACTCGGTCGCCGCATACACTATTCTC

AGAATGACTTGGTTGAGTACTCACCAGTCACAGAAAAGCATCTTACGGATGG

CATGACAGTAAGAGAATTATGCAGTGCTGCCATAACCATGAGTGATAACACTG

CGGCCAACTTACTTCTGACAACGATCGGAGGACCGAAGGAGCTAACCGCTTT

TTTGCACAACATGGGGGATCATGTAACTCGCCTTGATCGTTGGGAACCGGAG

CTGAATGAAGCCATACCAAACGACGAGCGTGACACCACGATGCCTGTAGCA

ATGGCAACAACGTTGCGCAAACTATTAACTGGCGAACTACTTACTCTAGCTTC

CCGGCAACAATTAATAGACTGGATGGAGGCGGATAAAGTTGCAGGACCACTT

CTGCGCTCGGCCCTTCCGGCTGGCTGGTTTATTGCTGATAAATCTGGAGCCGG

TGAGCGTGGGTCTCGCGGTATCATTGCAGCACTGGGGCCAGATGGTAAGCCC

TCCCGTATCGTAGTTATCTACACGACGGGGAGTCAGGCAACTATGGATGAACG

AAATAGACAGATCGCTGAGATAGGTGCCTCACTGATTAAGCATTGGTAACCG

ACGCCGACCAACACCGCCGGTCCGACGGCGGCCCACGGGTCCCAGGGGGGT

CGACCTCGAAACTTGTTTATTGCAGCTTATAATGGTTACAAATAAAGCAATAG

CATCACAAATTTCACAAATAAAGCATTTTTTTCACTGCATTCTAGTTGTGGTTT

GTCCAAACTCATCAATGTATCTTATCATGTCT
